# Randomized, double-blind, placebo-controlled clinical trial to assess the safety and effectiveness of a novel dual-action oral topical formulation against upper respiratory infections

**DOI:** 10.1186/s12879-016-2177-8

**Published:** 2017-01-14

**Authors:** Pranab K. Mukherjee, Frank Esper, Ken Buchheit, Karen Arters, Ina Adkins, Mahmoud A. Ghannoum, Robert A. Salata

**Affiliations:** 1 Center for Medical Mycology, University Hospitals Cleveland Medical Center and Case Western Reserve University, Cleveland, OH USA; 2 Division of Pediatric Infectious Diseases, University Hospitals Cleveland Medical Center and Case Western Reserve University, Cleveland, USA; 3 Division of Infectious Diseases and HIV Medicine, University Hospitals Cleveland Medical Center and Case Western Reserve University, Cleveland, OH 44106 USA

**Keywords:** Cetylpyridinium chloride, Glycerin, Xanthan gum, Antiviral, Barrier formation, Prophylaxis

## Abstract

**Background:**

Current prevention options for upper respiratory infections (URIs) are not optimal. We conducted a randomized, double-blinded, placebo-controlled pilot clinical trial to evaluate the safety and efficacy of ARMS-I™ (currently marketed as Halo™) in the prevention of URIs.

**Methods:**

ARMS-I is patented novel formulation for the prevention and treatment of influenza, comprising a broad-spectrum antimicrobial agent (cetylpyridinium chloride, CPC) and components (glycerin and xanthan gum) that form a barrier on the host mucosa, thus preventing viral contact and invasion. Healthy adults (18–45 years of age) were randomized into ARMS-I or placebo group (50 subjects each). The drug was sprayed intra-orally (3× daily) for 75 days. The primary objectives were to establish whether ARMS-I decreased the frequency, severity or duration of URIs. Secondary objectives were to evaluate safety, tolerability, rate of virus detection, acceptability and adherence; effect on URI-associated absenteeism and medical visits; and effect of prior influenza vaccination on study outcomes.

**Results:**

Of the 94 individuals who completed the study (placebo: *n* = 44, ARMS-I: *n* = 50), six presented with confirmed URI (placebo: 4, ARMS-I: 2), representing a 55% relative reduction, albeit this was statistically not significant). Influenza, coronavirus or rhinovirus were detected in three participants; all in the placebo group. Moreover, frequency of post-treatment exit visits was reduced by 55% in ARMS-I compared to the placebo group (*N* = 4 and 2, respectively). Fever was reported only in the placebo group. ARMS-I significantly reduced the frequency and severity of cough and sore throat, and duration of cough (*P* ≤ .019 for all comparisons). ARMS-I was safe, well tolerated, had high acceptability and high adherence to medication use. Medical visits occurred only in the placebo group while absenteeism did not differ between the two arms. Prior influenza vaccination had no effect on study outcome.

**Conclusions:**

This randomized proof-of-concept clinical trial demonstrated that ARMS-I tended to provide protection against URIs in the enrolled study participants, while reducing severity and duration of cough and sore throat. A clinical trial with a larger number of study participants is warranted.

**Trial registration:**

ClinicalTrials.gov NCT02644135 (retrospectively registered).

**Electronic supplementary material:**

The online version of this article (doi:10.1186/s12879-016-2177-8) contains supplementary material, which is available to authorized users.

## Background

Upper respiratory infections (URIs) are associated with significant morbidity and mortality, particularly in children, the elderly and those with underlying medical conditions (e.g. cancer, cardiopulmonary disease, diabetes and immunosuppression) [1–5]. The Centers for Disease Control (CDC) conducted a review of influenza cases over 31 influenza seasons (1976–2007) and reported that the annual rate of influenza-associated death in the US during this period ranged from 1.4 to 16.7 deaths per 100,000 persons [6]. Moreover, influenza is associated with 31 million hospital visits and >200,000 hospitalizations annually [2, 7, 8]. Infections associated with non-influenza viruses are known to cause 20 million lost work and school days annually, and yearly economic burden due to viral URIs ranges between $40 and $87 billion [9–11].

URIs are caused by respiratory viral pathogens including influenza, respiratory syncytial virus (RSV), human metapneumovirus, rhinovirus and adenovirus [7, 12, 13]. The current prevention strategies for influenza involve the use of vaccines and antiviral medications. Although vaccines are generally effective, their coverage and effectiveness vary (40–60%) [1, 14–17]. Other limitations of vaccinations include vaccine/strain mismatch and “vaccine hesitancy” [18, 19]. While neuraminidase inhibitors (NAIs, e.g. oseltamivir, zanamivir) are approved in the US to prevent and treat influenza [20, 21], these agents often induce only a modest decrease in symptom duration in people with uncomplicated illness [22–24]. NAIs can also be associated with resistance development, side effects, high cost and limited effectiveness [22, 24–30]. Therefore, an unmet need exists for the development of an effective therapeutic approach to prevent URIs.

ARMS-I (currently marketed as Halo™) was developed as a “first-in-class” novel dual-action formulation that can prevent viral URIs by killing the virus (by disrupting the host-derived viral lipid membrane) while forming a protective barrier on the host mucosa. Recently, our group demonstrated that the ARMS-I formulation exhibits novel potent activity against respiratory viruses in vitro [31], and reduced influenza-associated mortality and morbidity in an influenza infection murine model [32]. In the current study, we report on the safety and effectiveness of ARMS-I in preventing URIs in a randomized, double-blind, placebo-controlled pilot clinical trial.

## Methods

### Product description

ARMS-I is a single-stream oral spray that targets the oral oropharynx mucosal surfaces, and comprises a broad-spectrum antimicrobial agent (cetylpyridinium chloride, CPC) that disrupts the viral lipid envelope through physicochemical interactions, and components (glycerin and xanthan gum) that form a barrier on the host mucosa, thus preventing viral contact and invasion.

### Study design

The current study was a randomized, double-blinded, placebo-controlled pilot clinical trial. The hypothesis of the study was that the use of the active product (ARMS-I) sprayed intra-orally 3× daily is associated with fewer episodes and a lower duration and symptom severity of acute URIs. Acute URIs were defined as a combination of three of any of the following symptoms: fever (≥37.8 °C), non-productive cough, sore throat, rhinorrhea (runny nose), sinus congestion (stuffy nose) and malaise [33]. The enrollment target was 100 healthy men and women (18–45 years old, inclusive). Health of study participants was assessed based on patient’s recall of symptoms and clinical assessment (inclusion criteria: BMI of 17–35 kg/m^2^, no tobacco/nicotine use for at least 3 months, and non-pregnant or breast-feeding; for all inclusion/exclusion criteria and study design details, see protocol in Additional file 1). Participants were enrolled into the study after informed consent following a clinical trial protocol approved by the University Hospitals Case Medical Center Institutional Review Board for Human Investigation, Cleveland, OH (protocol number 11-11-33, approval date: 12/16/2012, for details regarding background information about the eligible participants and eligibility criteria, see full protocol in Additional file 1). All subjects provided written consent, obtained in accordance with Federal Regulations, and were compensated monetarily for their participation. The written document embodied the elements of informed consent as described in the Declaration of Helsinki and adhered to the ICH Harmonized Guideline for Good Clinical Practice. The clinical trial protocol went through a rigorous review at our Institutional Review Board, and adhered to all components necessary for a pilot clinical study of this nature. Moreover, the protocol was also carefully vetted by ClinicalTrials.gov during registration process (NCT02644135), which did not indicate any deficiencies in trial design. There are no additional currently ongoing clinical trials with this product.

Study participants were randomized with equal proportion (50 each) into two groups: (a) active product (ARMS-I) administered intra-orally by spray three times daily (dosing regimen selected based on a pilot clinical study evaluating the ability of ARMS-I to reduce oral microbial load [31, 34, 35]) or (b) placebo (purified sterile water containing the same flavor as the active but without neither the active antimicrobial agent (CPC) nor the barrier forming components) administered intra-orally by spray three times daily. Randomization lists were generated using the website https://www.randomizer.org/, and all study personnel except the pharmacist were blinded. The active or placebo agents were self-administered daily by participants for 75 days. An exit visit occurred within 2 weeks post-treatment. The study was conducted during the 2013 respiratory virus season in Northeastern Ohio (date range for patient recruitment was January 8, 2013 through March 23, 2013), completed on June 17, 2013 and unblinded on July 22, 2013.

The primary objectives were: (1) to determine whether ARMS-I decreases the frequency of acute URIs, and (2) to assess whether ARMS-I decreases the duration and severity of URI-related symptoms. Secondary objectives were: (1) to assess the tolerability, acceptability and adherence to ARMS-I medications vs. placebo, (2) to compare whether acute URIs in those receiving ARMS-I compared to placebo are associated with differences in absenteeism (from work or school) and visits to physicians’ offices, emergency departments and urgent care centers, (3) to determine whether ARMS-I decreases the detection of respiratory viruses by polymerase chain reaction (PCR) [36–39], and (4) to evaluate the effect of ARMS-I on those who did or did not receive the influenza vaccine. The endpoints were: (1) frequency and duration of clinical respiratory disease at study visits and as assisted by electronic patient diaries, (2) intra-and extra-oral exams, (3) solicited and unsolicited adverse events, (4) respiratory virus multiplex PCR, and (5) self-report for adherence to medication usage. Subject-level characteristics were summarized per study group, age, gender, prior influenza vaccine status and medications taken for symptom relief. The study length and number of study surveys completed were used to summarize the information on the frequency, duration and severity of symptoms.

### Assessment of symptoms

Severity of URI-related symptoms was scored on a 5-point scale (0 = None, 1 = Minor, 2 = Mild, 3 = Moderate, 4 = Severe), based on diary entries from study participants with at least three symptoms using the validated Wisconsin Upper Respiratory Symptom Survey (WURSS-21) [33]. Determination of duration of URIs-related symptoms was performed by assessment of self-reported diaries of study participants (with at least three symptoms) to identify instances where the symptoms were present for at least two consecutive days. Frequency of symptoms occurring at least 1 week apart were recorded as distinct occurrences (since multiple URI events can occur per individual in a season). Participants with a diary-based URI event were asked to present at the clinic, where the study physicians examined them to confirm clinical URI.

### Data collection

We used the Research Electronic Data Capture (REDCap) method to collect, store and disseminate trial-specific clinical data [40]. Electronic diaries were created using the REDCap system, and participants recorded their symptoms and addressed the study-related questionnaire using these electronic diaries. Data analysis was performed to address the primary and secondary objectives described in the study design. Frequency of URI was assessed based on: (1) visits to the clinic where the study participant had at least three URI-related symptoms (“sick visits”, confirmed clinically by study staff), (2) interviews conducted by study nurses with the study participants within 2 weeks of treatment completion and (3) analysis of daily diaries electronically completed by study participants, describing the presence of at least three symptoms.

### Sample size calculations

Sample size calculations were carried out assuming that the two groups would be of equal size and that the random assignment would be balanced. Further, it was assumed that an average of two events would occur in the control group, compared to an average of 1.5 events in the treated group (Mean ± SD = 1.5 ± 1.0), and that the average duration of illness would be 4 days and 3 days in the control and treated groups, respectively (Mean ± SD = 4 ± 2 and 3 ± 2, respectively). Taking an alpha to be 0.05, a sample size of 23 per group would allow the detection of a 25% difference in primary outcomes between the two groups with 80% power. The sample size was increased to 50 per group to account for potential losses to follow-up.

### Statistical analyses

Each symptom of URIs was investigated separately. For each endpoint, the total number of days for which there was an event was recorded. Then, the number of days for which there was an event per 75 days of person-time follow-up (related to the study duration per subject) was recorded in each group. Since this is a prevention study conducted during the flu season of 2013, we selected 75 days so that we covered the entire season. Next a logistic regression model was constructed. The data were taken at the day level, so the endpoint is yes/no for an event on that day. Furthermore, the data included every day for which there was a completed survey. The endpoints were assessed for each treatment group (placebo vs. ARMS-I). Because there were multiple daily observations for each individual in the study (nominally 75 repeated measures per subject, but different for each subject) an ordinary logistic regression model was inappropriate because the observations within a subject from day-to-day would be expected to be correlated. Therefore, generalized estimating equations were used to fit the regression model. Medical visits (an indicator of whether a subject went to an Emergency Department, an urgent care center or a doctor’s office due to URIs symptoms on each day) and absenteeism (an indicator of whether a subject missed school or work or would have missed school or work if it were scheduled on each day) were analyzed the same way as the individual symptom analyses. The effect of vaccine status on the outcomes was assessed by fitting a multivariable logistic regression model with the treatment arms and vaccine status as the explanatory variables.

## Results

### Subject demographics

**Fig. 1:**
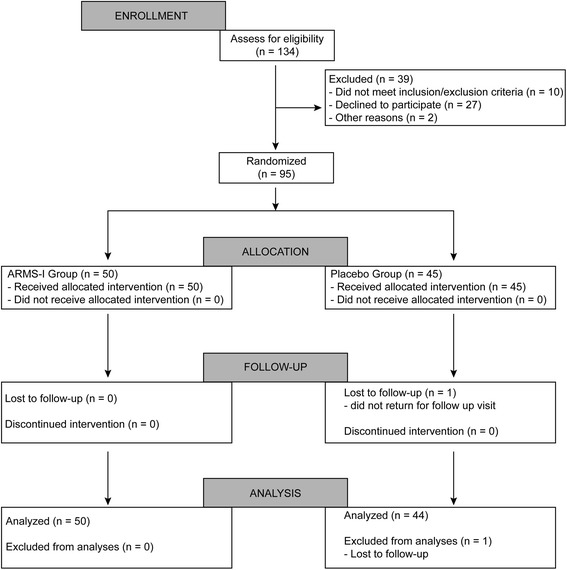
CONSORT flow figure for the conducted study

A total of 100 individuals were enrolled and randomly assigned to the two treatment arms, of which five did not begin the study. One subject (in placebo group) did not return for follow-up visit, and was excluded from the analyses. Thus, analysis of results was performed for 94 participants, of whom 44 were in the placebo group and 50 were in the ARMS-I group (see Fig. 1 and Additional File 2 for CONSORT checklist).

Table 1 summarizes the demographics of study participants. The age of study participants ranged between 18 and 43 years in both groups, with the mean age of 24.86 ± 6.47 years in the placebo group and 25.14 ± 6.73 years in the active group, with no significant difference observed between the two groups (*P* = .68). The gender distribution was also similar in the two study groups, with 24 males and 20 females in the placebo group (54.5 and 45.5%, respectively) and 24 males and 26 females in the active group (48 and 52%, respectively). The study duration (number of days from enrollment until the 3^rd^ follow-up visit) was similar with 72.8 ± 2.9 for the placebo group and 71.8 ± 2.8 for the ARMS-I group. There were a total of 5945 surveys completed in the study (2849 in the placebo group and 3096 in the active group). Moreover, percent surveys completed (number of surveys completed divided by study duration) were similar in both groups; 89.1 ± 15.6% and 86.3 ± 20.6% for the placebo and active group, respectively.

**Table 1:** Summary of study demographics

Variable	Placebo	Active	*P*-value
Total enrolled	44	50	-
Male	24 (54.5%)	24 (48.0%)	1.0^*^
Female	20 (45.5%)	26 (52.0%)	.466^*^
Age (Mean ± SD)	24.86 ± 6.47	25.14 ± 6.73	.807^†^
Age range	18-43	18-43	

^*^Chi square test, asymptotic significance

^†^Independent samples t-test

### Frequency of acute URIs in active and placebo groups

Among the 94 enrolled individuals, there were six participants who presented to the clinic for clinical confirmation and collection of oral and nasal swabs related to the development of URIs symptoms (Confirmed URIs Episode). Of the participants who presented with a Confirmed URI episode, four (9%) belonged to the placebo and two (4%) belonged to the active group (95% CI 0.0725, 2.3941, *P* = .41), indicating a relative reduction of 55% in the latter. Moreover, six (additional) subjects reported URI-related symptoms at their post-treatment visit (within 2 weeks of study completion); among these individuals, four were in the placebo group and two were in the active group. Analyses of diary entries showed that one additional subject in the active group and four additional subjects in the placebo group recorded symptom-based URIs. These analyses showed that the cumulative frequency of URIs tended to be lower in individuals using ARMS-I than those using the placebo.

### Effect on frequency, duration and severity of URI symptoms

Analyses of the symptoms reported by study participants in their daily diaries showed a total of 64 occurrences of URIs, observed in 20 individuals. Among these, 37 occurred in the placebo group (in 11 individuals) while 27 occurred in the active group (in nine individuals). The frequency of URIs tended to be higher in individuals in the placebo group than those in the active group (25% vs. 18%, respectively, OR = 1.4, 95% CI: 0.635–3.037), indicating a 28% lower relative frequency of URIs in the active group. Analysis of the data based on daily surveys (events) of symptoms also revealed a similar pattern with a 44% relative reduction in the frequency of URIs in the active group, although this difference was not significant (OR = 1.5, 95% CI: 0.909–2.439).

Analysis of severity of URIs showed that while fever was reported only in the placebo group (10.8%), frequency of cough and sore throat were significantly reduced in the active group (Table 2, *P* ≤ .008). Moreover, severity of cough and sore throat were also significantly reduced in the active group compared to placebo group, while frequency of stuffy nose was significantly increased in the active group (*P* ≤ .001, Table 2).

**Table 2:** Frequency and severity of diary-based symptoms in study participants with upper respiratory infections

Symptom	Frequency (%)^*^			Severity (mean ± SD)		
	Placebo	Active	*P*-value	Placebo	Active	*P*-value
Cough	29 (78.4%)	7 (25.9%)	< .001	1.73 ± 1.36	0.56 ± 1.01	< .001
Sore throat	30 (81.1%)	13 (48.1%)	008	1.73 ± 1.19	0.74 ± 0.85	.001
Runny nose	25 (67.6%)	18 (66.7%)	1	0.95 ± 0.88	1.56 ± 1.28	.027
Stuffy nose	19 (51.4%)	26 (96.3%)	<.001	0.89 ± 1.05	2.07 ± 0.87	<.001
Malaise	22 (59.5%)	21 (77.8%)	.179	1.49 ± 1.38	1.67 ± 1.03	.572
Fever	4 (10.8%)	0	-	100 – 103 °F	-	

^*^Percentage values are compared to the total number of events

Chi-square analysis of symptoms in individuals with URIs showed that the relative risk of cough in the placebo group was 3-times that of people in the active group, while the relative risk of sore throat was 1.6-times that of people in the active group (Table 3, *P* ≤ .008). Furthermore, multivariable logistic regression analysis indicated that cough was the only symptom that associated significantly with URI, with less cough in the active group (placebo vs. active, *P* = .012; 95% CI: 0.019–0.606).

**Table 3:** Chi-square analysis of symptoms in individuals with URI, in the active and placebo arms

Variable	Relative risk (RR)	Odds ratio (OR)	95% CI for RR
Cough	3.02	0.10	1.563 - 5.847
Sore Throat	1.68	0.22	1.105 - 2.566
Runny Nose	1.01	0.96	0.716 - 1.435
Stuffy Nose	0.53	24.63	0.386 - 0.736
Malaise	0.76	2.39	0.548 - 1.067

We assessed the duration of symptoms in individuals who reported URIs-related symptoms for at least two consecutive days. Fever was reported only in the placebo group with duration of 2 days. The median duration of cough, sore throat or runny nose was 2.5 days for each in the placebo group, while the median duration of these symptoms was 0, 1 or 2 days, respectively, in the ARMS-I group. The median duration of stuffy nose and malaise was 2 days in both study groups. The maximum duration for all the non-fever symptoms was between 5 and 9 days in the placebo group, while this duration was lower (3-5 days) in the active group (*P* = .019 for cough, >0.05 for all other comparisons).

### Safety, tolerability, acceptability and adherence to use of ARMS-I

The safety, tolerability, acceptability and adherence were evaluated by oral exams, solicited and unsolicited adverse events (AEs), end-of-study acceptability surveys and self-reported use of sprays. As part of the study protocol, oral exams were conducted on all study participants. Among the 94 enrolled participants, abnormal oral exams were reported for four individuals, of which three belonged to the placebo group (cheek biting for two, and labial mucosal injury in one participant) and one was in the active group (enlarged tonsils at enrollment, not noted at subsequent visits or at end of study). None of these oral events were considered related to the study drug. A total of nine adverse events (AEs) were reported in the study (with a 75-day duration), of which five occurred in the placebo group (headache, two; anxiety, labial mucosal injury and muscle strain, one each), while four occurred in the active group (headache, two; anxiety and extremity rash, one each). None of the AEs were considered related to the study medication.

Participants were asked to complete an exit questionnaire with questions related to acceptability of the active product at the end of the study. We found that 60% of the respondents “strongly liked” or “liked” the taste of the active product, while 27.5% were “neutral”. Moreover, 95% of the respondents had a favorable opinion about the smell (35% “strongly liked” or “liked”, 65% were “neutral”) of the product. In addition, 79.2% of the participants stated that they would recommend the product to others, while a majority (56.3%) expressed willingness to continue to use the product after the study ended. These results demonstrated that ARMS-I had high acceptability among the study participants. Our analysis showed that the single-stream spray bottle was used as indicated in ≥85% of the days in the placebo and ≥86.9% in the active group. These results indicate that study participants exhibited a high degree of compliance applying the study drug 3 times a day.

### Effect of URIs on absenteeism and hospital visits in the active and placebo groups

There were a total of 5945 surveys completed in the study (2849 in placebo and 3096 in active group). The medical care question was left blank on 61 surveys, thus data is only available for 5884 surveys (2841 in placebo and 3043 in active group). Among individuals with URIs, there were two medical visits, both in the placebo arm, and nine absenteeism of which five (13.5%) were in the placebo group, and four (14.8%) in the active group. These results showed that medical visits occurred only in the placebo group while absenteeism did not differ between the two arms.

### Frequency of respiratory viruses

PCR analysis performed on the oral and nasal swabs collected from individuals with URIs showed the presence of influenza B, coronavirus or rhinovirus (OC43) in three participants (detected in February, March and April, respectively). All three infected participants belonged to the placebo arm.

### Effect of vaccination status

Among the enrolled 94 individuals, 41 reported receiving influenza vaccine previously, of which 17 (38.6%) belonged to the placebo group while 24 (48%) belonged to the active group. Multivariable logistic regression analysis revealed the vaccine status had no significant effect on URIs (*P* = .15). These results showed that vaccination status did not influence the URIs between the two arms.

## Discussion

In the current study, we evaluated the safety and effectiveness of ARMS-I, a novel intra-oral formulation in the prevention of URIs in a randomized, double-blind, placebo-controlled proof-of-concept clinical trial in healthy adults. Our data showed that the product is safe and well tolerated, and it reduces symptoms associated with influenza. Use of ARMS-I was associated with a trend to reduced frequency of URIs.

Our study demonstrated that ARMS-I was safe and had no drug-related adverse effects. This is to be expected, based on the known safety profile of the active ingredients and their long history of use in humans [41–46]. The novel, patented ARMS-I formulation contains cetylpyridinium chloride (CPC) as an antimicrobial, and xanthan gum and glycerin as the barrier forming agents. These ingredients have been used since the 1940s as components of various drug products including oral sprays, tablets, lozenges and capsules at concentrations similar to those present in ARMS-I [43].

Two randomized, double-blind clinical trials have reported the efficacy of orally administered agents in the prevention of URIs. O’Neil et al. [47] compared the efficacy of commercially available *Echinacea* capsules in preventing URIs symptoms compared to placebo over a period of 8 weeks during the winter months, and reported that *Echinacea* capsules did not significantly alter the frequency of URIs symptoms. Bennett et al. [48] determined the efficacy of low dose interferon alpha (IFN-α) lozenges in the prevention of URIs in healthy adults (*n* = 275, aged 18–75 years), based on weekly health data questionnaires. These investigators reported that low-dose oral IFN-α prophylaxis did not affect the incidence of URI, but did reduce the severity and duration of symptoms.

Our study showed that oral topical administration of the active agent was associated with a trend of frequency reduction, and significantly reduced severity and duration of cough and sore throat associated with URIs. Interestingly, severity of runny nose increased significantly in the active group, as did frequency and severity of stuffy nose, which could be linked to the fact that the product is applied orally and not intranasally. In this regard, Lakdawala et al. [49] identified soft palate of the oropharynx as an important site of isolation of transmissible virus and an initial site of infection. Thus, drugs like ARMS-I, that target the oropharynx, could represent an novel approach for the prevention of viral respiratory infections.

ARMS-I possesses a dual mechanism of action that: (a) targets the host by forming a barrier that prevents contact between the virus and the host mucosa, and (b) exerts direct virucidal activity that disrupts the outer viral membrane [31, 32]. Since CPC, the antiviral component of ARMS-I, targets host-derived lipid membrane through physicochemical interactions and does not target a viral protein, activity of ARMS-I is unlikely to be affected by mutations in the viral genome. Thus, ARMS-I has the additional advantage of having a low potential for the development of resistance.

Limitations of the current study include being under-powered, and the low incidence of URIs in the cohort, which may be due to the seasonal nature of URIs, as well as participants who recorded URIs in their diaries but did not present at the clinic. Other potentially confounding variables include ethnicity, occupational status and co-morbidity of chronic respiratory diseases. In future planned investigations, we intend to power the clinical trial based on the low incidence of URIs as well as conduct the study over multiple sites, and multiple seasons.

## Conclusions

ARMS-I is safe and well-tolerated, and it reduces influenza symptoms. This product has the potential to prevent viral upper respiratory tract infections. Further clinical development of this novel product is warranted.

## Additional files

Additional file 1: Trial ProtocolR2. (DOCX 7895 kb)
Additional file 2: CONSORT 2010 ChecklistR2. (DOC 216 kb)
Additional file 3: RawDataR2. (XLSX 669 kb)

## Abbreviations

AEs: Adverse events
CPC: Cetylpyridinium chloride
ICH: International Council for Harmonisation of Technical Requirements for Pharmaceuticals for Human Use
IFN-α: Interferon alpha
NAIs: Neuraminidase inhibitors
PCR: Polymerase chain reaction
REDCap: Research Electronic Data Capture
RSV: Respiratory syncytial virus
URIs: Upper respiratory infections
WURSS: Wisconsin Upper Respiratory Symptom Survey
