# Supplementary material for: Randomized, double-blind, placebo-controlled clinical trial to assess the safety and effectiveness of a novel dual-action oral topical formulation against upper respiratory infections
Source: BMC Infect Dis. 2017 Jan 14;17:74. doi: 10.1186/s12879-016-2177-8 (PMC5237564; doi:10.1186/s12879-016-2177-8)
Supplement: Additional file 1: — Trial ProtocolR2. (DOCX 7895 kb) [file 12879_2016_2177_MOESM1_ESM.docx]

**Pilot Study of the Safety, Tolerability, and Effectiveness of Halo to Prevent Acute Upper Respiratory Illness and Respiratory Virus Infections**

**Version 3.1 11/26/2012**

**Investigators**

**Principal Investigator**

Robert A. Salata, MD, Professor and Executive Vice-Chair, Department of Medicine, Chief, Division of Infectious Diseases and HIV Medicine, Case Western Reserve University, University Hospitals Case Medical Center

**Co-Principal Investigator**

Frank Esper, MD, Assistant Professor, Department of Pediatrics, Division of Infectious Diseases and Rheumatology, Case Western Reserve University, University Hospitals Case Medical Center

**Co-Investigators**

Pranab K. Mukherjee, PhD, MSc, Assistant Professor, Center for Medical Mycology/Department of Dermatology, Case Western Reserve University

Richard J. Jurevic, DDS, PhD, Assistant Professor, Biological Sciences, School of Dental Medicine, Case Western Reserve University

**Sponsor**

Oasis Consumer Healthcare, LLC, 812 Huron Rd., Suite 235, Cleveland, OH 44115

**Protocol Schema**

A Pilot Study of the Safety, Tolerability, and Effectiveness of Halo to Prevent Acute Upper Respiratory Illness and Respiratory Virus Infections

**DESIGN** This is a randomized, double-blinded, placebo-controlled clinical trial

**DURATION** Participants will be on treatment for 75 days. A follow up visit will occur within 2 months after treatment ends.

**SAMPLE SIZE** 51 participants each in the treatment and placebo arms

**POPULATION** Healthy men and women 18 to 45 years old, inclusive

**INTERVENTION** Participants will be randomized with equal proportion (51 each) into one of the following 2 groups:

- Halo administered intra-orally by spray Three times daily
- Placebo administered intra-orally by spray Three times daily

**HYPOTHESIS** The use of Halo sprayed intra-orally three times daily

will be associated with fewer episodes and duration of acute

respiratory diseases and less frequent detection of important

respiratory viruses confirmed by multiplex PCR

**PRIMARY OBJECTIVES** 1. To establish whether Halo will decrease the

frequency of acute upper respiratory illness

2. To assess whether Halo will decrease the duration

and severity of acute upper respiratory illness

**SECONDARY**  1. To assess the tolerability, acceptability and adherence to

**OBJECTIVES** Halo

2. To compare whether acute upper respiratory disease in

those receiving Halo compared to placebo is associated with differences in absenteeism (from work or school) and visits to physicians’ offices, emergency departments and urgent care centers

3. To determine whether Halo will decrease

the detection of respiratory viruses by PCR

4. To evaluate the effect of Halo on bacterial and fungal

microflora in the oropharynx

5. To evaluate the effect of Halo on those who did and did not receive the influenza vaccine.

**ENDPOINTS** 1. Frequency and duration of clinical respiratory disease at

interim visits and assisted by patient diaries

2. Intra-and extra-oral exams

3. Solicited and unsolicited adverse events

4. Respiratory virus multiplex PCR

5. Bacterial (oral streptococci, Group A streptococcus) and

fungal cultures

6. Bottle counts and self-report for adherence

**Significance and Background**

Respiratory tract disease is a major cause of morbidity and mortality throughout the world accounting for approximately 4 million deaths worldwide [[1](#_ENREF_1), [2](#_ENREF_2)]. Asthma, pneumonia, and acute bronchiolitis account for almost 22% of all pediatric hospitalizations in the United States. Pneumonia remains in the top ten causes for admission across all age groups in the United States [[3](#_ENREF_3)]. Much of which are directly caused by, or triggered from respiratory viruses including influenza, parainfluenza, respiratory syncytial virus (RSV), and adenovirus. In addition to these well-known pathogens, advancements in molecular and genomic techniques have increased our detection and identification of new respiratory viruses which also contribute to respiratory disease [[4-13](#_ENREF_4)]. There is also renewed attention on respiratory viruses previously considered benign, including the human rhinovirus and coronaviruses, as there is mounting evidence that these viruses can also lead to clinically severe disease [[6](#_ENREF_6), [14-20](#_ENREF_14)]. Influenza is one of the leading causes of infectious related deaths and hospitalizations in children and the 2009-10 H1N1 pandemic was especially severe in younger populations [[21](#_ENREF_21), [22](#_ENREF_22)].

Respiratory disease can be caused by a variety of micro-organisms especially viruses. Despite the frequency and burden of acute respiratory infections worldwide and across all age groups, there has been limited progress in the management of treatment of respiratory virus infections aside from the use of anti-influenza drugs. The ability to prevent of acute respiratory disease has also been more limited except for the use of the influenza vaccine. The availability of a product that can be used with ease, is low cost but possesses broad spectrum antimicrobial and antiviral activity could play a major role in reducing the overall occurrence, morbidity and mortality associated with acute respiratory disease.

Moreover, upper respiratory infections are the leading cause of outpatient illness in U.S. military professionals and serve as the single greatest cause of lost workdays in the military [[23](#_ENREF_23)]. Unfortunately, these infections are not only prevalent, but also severe, constituting a primary cause of infectious disease hospitalization in U.S. military personnel including Navy shipboard and submarine personnel [[23](#_ENREF_23), [24](#_ENREF_24)], and new recruits at basic training facilities [[25](#_ENREF_25), [26](#_ENREF_26)]. Military trainees and newly-mobilized troops are particularly subject to upper respiratory infection epidemic outbreaks due to their crowded living conditions, stressed working environments, and exposure to respiratory pathogens in disease-endemic areas. Additionally, the pulmonary disease resulting from ubiquitous infections are particularly impactful in high altitudes and other extreme environments common to Air Force/Navy activities [[24](#_ENREF_24)].

Oasis Health Care has developed a technology and product that combines microbial barrier abilities along with a broad spectrum anti-infective agent cetylpyridinium chloride (CPC) at 0.1%. This formulation called Halo is commercially available for the over-the–counter treatment of xerostomia. The spray formulation has high mucoadhesion properties and prolonged retention time in the oral cavity (21). Cetylpyridinium chloride like chlorhexidine is a quarternary ammonium salt. However, due it possesses certain advantages over the latter in that it targets pathogenic bacteria while sparing beneficial ones (see below). Due to its chemical structure, CPC demonstrates significant antiviral activity especially against respiratory viruses (see below). Toxicology studies have shown no evidence of acute or chronic toxicity in animal models or irritant effect, and over-the-counter mouthwashes containing cetylpyridinium chloride have been marketed for over 45 years. Expanding the indications for the use of a cetylpyridinium-based spray formulation (Halo) as a potential formulation to prevent respiratory virus infections (cold and flu) is the long term goal. This proposal will examine the effect of Halo on the frequency and duration of clinically apparent acute respiratory infections and on respiratory virus burden as measured by PCR in young healthy adults through the respiratory virus season in Cleveland, Ohio.

**Preliminary Studies**

**Activity against respiratory viruses**

**Halo inhibits the infectivity of influenza A**

The antiviral activity of Halo was evaluated employing a clinical strain of influenza A (2009/H1N1/infA) as a prototype virus.

We examined the ability to inhibit the infectivity of influenza A. Madin Darby canine kidney (MDCK) Cells were prepared in 24-well plates with Dulbecco's Modified Eagle Medium (DMEM) with 10% heat inactivated fetal calf serum and supplemented with L-glutamine (Lglu) and penicillin/streptomycin (P/S) (unless specified, all reagents produced by Gibco, N.Y., USA). All culture cells were grown to 90-100% confluence at 37° at 5% CO2 prior to infection. Halo compound was applied to cell monolayers at 20% concentration (100ul 1X Halo to 400ul optiMEM (+P/S, +Lglu)) for a working CPC concentration of 0.02%. Halo suspension or control (500ul optiMEM (+P/S,+Lglu) was applied to cells at Intervals: T-2hours, T-1hour, T-30min, and T0 (Immediate) prior to infection. At time of infection, Media containing OralGuard were removed from cells. Cell monolayers were infected with influenza A at multiplicity of infection ( MOI) of 0.1. Cells were then centrifuged @ 2200rm X 30min and 500ul of optiMEM (+P/S, +Lglu, 2ug/ml trypsin (sigma-Aldrich, St Louis, MO)) was applied. Infected cells were grown at 32.5° X 48 hours at 5%CO2.

Our data showed that at 48 hours post-infection, cell monolayers treated with Halo compound at T-2hours, T-1hour, and T-30min remained confluent and healthy. Untreated cells and cells treated immediately prior to infection (T0) demonstrated substantial cytopathic effect (Figure 1).

To examine the effect of Halo on influenza, cells were removed and fixed to 10well Teflon printed slides (EMS, Hatfield , PA) with 80% acetone. FITC labeled anti-influenza antibody (D^3^ Ultra Flu-A, Diagnostic Hybrids, Athens, OH) was applied and incubated for 1 hour. As seen in Figure 2, fluorescence was observed in all untreated controls. In contrast, no fluorescence was observed in Halo treated cells at T-2hours, T-1hour, T-30min timepoints.


To quantitate the effect of Halo on influenza virus, cell culture supernatants were collected and stored at -80°. Nucleic acid were extracted using QIAamp Viral RNA Kit (QIAGEN, Valencia, CA). Random hexamer primers (Invitrogen Carlsbad, CA) were used to create a cDNA library for each specimen. Reverse transcription reactions were performed with M-MLV RT (Invitrogen, Carlsbad, CA) according to the manufacturer's specifications. Quantitative analysis was performed on a StepOne Plus Taqman Real Time PCR (Applied Biosystems, Branchburg, NJ ) using TaqMan Universal PCR Master Mix (Applied Biosystems, Branchburg, NJ) , 2µl of cDNA sample, and primers/probes targeting the influenza matrix gene. A reference standard was prepared using a cDNA fragment of the H1N1 matrix gene and human RNAse P amplified by conventional RT-PCR, gel purified (QIAquick, Qiagen, Valencia, CA), and quantified using a spectrophotometer (Beckman Coulter, Brea, CA). Halo treated cells at T -2hours, T -1hour, T-30min timepoints did not have detectable influenza at 48hours post infection (Figure 3). Cells treated immediately prior to infection (T0) had an influenza viral load similar to that of untreated cells.

**Simulating physiologic removal of Halo**

We examined the ability to inhibit the infectivity of influenza A while simulating the removal of the Halo compound. African Green Monkey Kidney (CV-1) cells were grown in 24-well plates to 90% confluence as described above. Next, Halo compound was applied to cells (20% Oasis, 80%OptiMeM, working CPC concentration of 0.02 %.). Each timepoint matched with control (No compound applied, 100% OptiMeM). Compound was allowed to dwell on surface for 30 minutes. The Halo compound was then removed from the cell monolayer. Cells were then thoroughly washed twice with sterile optiMEM (+P/S,+Lglu). Influenza was inoculated at MOI = 0.1 at 30 minute intervals from T0 through T + 6hours. Following infection cells were then centrifuged @ 2200rm X 30min and 500µl of optiMEM (+P/S,+Lglu, 2µg/ml trypsin (sigma-Aldrich, St Louis, MO)) was applied. Infected cells were grown at 32.5° X 96 hours at 5%CO2. The influenza viral load was determined by real time PCR as described above.

Our study showed that despite removal of the Halo compound, a substantial reduction in influenza viral load was observed (figure 4). The inhibition of influenza infectivity appeared to remain constant between 0 and 6hours.

**Optimal CPC concentration for anti-viral properties**

We examined inhibition of influenza A infectivity by Halo at different concentrations to determine the most effective dosage. CV-1 cells were prepared as described above. Halo compound was applied to cells at 5%, 10%, 15%, and 20% in optiMEM (+P/S,+Lglu). Final working CPC concentrations were 0.005%, 0.01%, 0.015%, 0.02% CPC, respectively. Each concentration was matched with control (No compound applied, 100% OptiMeM). Halo compound was applied at T-2hours, T-1hour, and T0 (immediate) prior to infection and then thoroughly removed and washed twice with OptiMeM. Influenza virus was inoculated as described above. Infected cells were grown at 32.5° X 96 hours at 5%CO_2_. Influenza viral load determined by real time PCR as described above.

Influenza inhibition by Halo was greatest at 20% concentration (0.02% CPC) (Figure 5). No anti-infective activity was appreciated at 5 or 10% (0.005%, 0.01% CPC). It is possible that greater concentrations of Halo (i.e. 30%, 40%) may result in greater reduction in influenza titers.

**Halo vs. placebo**

To determine if the anti-infective effect demonstrated by Halo is attributed to the CPC compound or another reagent within the formulation (preservative), the effect on influenza infection was compared between Halo compound (With CPC), Compound without CPC but with preservatives, placebo alone (without CPC and preservatives), or media alone (optiMEM (+P/S,+Lglu). CV-1 cells were prepared as described above. Halo compound, Placebo with preservatives, and placebo alone were applied to cells at T-2hours, T-1hour, and T0 (Immediate) prior to infection and then thoroughly removed and washed twice with OptiMeM . Influenza virus was inoculated as described above. Infected cells were grown at 32.5° X 96 hours at 5%CO_2_. Cytopathic effect, immunofluorescence and influenza viral load determined by real time PCR were performed as described above.

Our data showed that cell monolayers treated with Halo compound remained confluent and healthy. In contrast, controls without Halo, and those with placebo with preservative, placebo without preservative demonstrated substantial cytopathic effect (Figure 6).

Substantial fluorescence was observed in all untreated controls, cells treated with placebo with preservative but without CPC, placebo without preservative or CPC, and media alone. Scant fluorescence was observed in Halo treated cells (figure 7).

Significant decrease in influenza viral load was again seen with cells treated with Halo (Figure 8). A 1.0 log decrease was also seen with the placebo without CPC but containing preservative. This likely represents some effect from the preservative. Placebo and media demonstrate no effect in viral titers.

**Halo has direct antiviral effect against influenza**

To determine if Halo has direct antiviral properties against influenza, we pretreated the influenza virus with Halo prior to infection and then diluted away the Halo product. CV-1 cells were prepared as described above. A standardized amount of influenza (0.1 MOI) was pretreated with 5%, 20%, or 100% Halo compound (containing 0.005%, 0.02%, 0.1% CPC respectively), compound without CPC but with preservatives, or placebo alone (without CPC and preservatives) for 5 minutes at room temperature. After the 5 minute incubation virus/drug mix was diluted by an additional equal volume with optiMEM (+P/S,+Lglu) to dilute out the Halo compound . Halo treated and untreated virus was then inoculated onto cells as described above. Influenza viral load determined by real time PCR was determined as described above.

Our data showed significant decrease in influenza viral load with virus pretreated with Halo (Figure 9). Further analysis will need to be performed to determine if longer incubation generate enhanced antiviral activity. Interestingly, compounds within the Halo formulation also appear to have direct anti-influenza activity to a lesser degree

**Antibacterial and antifungal activity of Halo**

The antimicrobial activity of Halo was evaluated against a panel of aerobic and anaerobic bacteria using M07-A8 and M11-A7methods respectively, as described by the Clinical and Laboratory Standards Institutes (CLSI). A standardized inoculum of bacteria (1 x 10^4^ cells/mL) was incubated with serially diluted solutions of Halo (0.1% CPC, or 1 µg/mL) or chlorhexidine (2% chlorhexidine gluconate, or 20 µg/mL) as comparator. Cells were allowed to grow in the presence or absence (growth control) of the test agents for 24 h. The MIC for each agent was defined as the concentration that induced a 100% growth inhibition (compared to no-drug control). As can be seen in Table 1, Halo exhibited potent activity against *S. salivarius*, *P. gingivalis, S. pyogenes, S. pyogenes, S. pneumonia* (MIC = 0. 98 µg/mL for all), *F. nucleatum* and *S. mutans* (MIC = 1.95 µg/mL against both). The MIC of Halo against *S. oralis* and *S. mitis* was noticeably elevated (500 µg/mL) compared to other organisms. It is interesting to note that *S. oralis* and *S. mitis*, are normal commensals of the oral cavity. In contrast to Halo™, chlorhexidine exhibited activity against both pathogenic as well as commensal oral organisms (MIC = 19.6 µg/mL).

We used a similar microdilution-based CLSI method (M27-A2) to evaluate the activity of Halo against *albicans* and non-*albicans* *Candida* species. Our results showed that the MIC (50% inhibition of growth) of Halo against *C. albicans*, *C. krusei*, *C. tropicalis*, or *C. glabrata* ranged between 0.06 µg/mL and 0.25 µg/mL), while the MIC of chlorhexidine was much higher (19.6 µg/mL, see Table 1). Taken together, these results demonstrate that Halo possesses potent activity against pathogenic bacteria and fungi commonly isolated from the oral cavity. This activity was more potent than that observed for chlorhexidine.

**Table 1. Minimum inhibitory concentration (MIC, µg/mL) of Halo and cholrhexidine against bacteria and fungi**

| **Organism** | **Halo**  **(µg/mL CPC)** | **Chlorhexidine**  **(µg/mL chlorhexidine)** |
| --- | --- | --- |
| ***S. salivarius*** | 0.98 | 19.6 |
| ***P. gingivalis*** | 0.98 | 19.6 |
| ***S. pyogenes*** | 0.98 | 19.6 |
| ***S. pneumoniae*** | 0.98 | 19.6 |
| ***F. nucleatum*** | 1.95 | 19.6 |
| ***S. mutans*** | 1.95 | 19.6 |
| ***S. aureus*** | 3.91 | 19.6 |
| ***Y. enterocolitica*** | 3.91 | 19.6 |
| ***S. oralis*** | 500 | 19.6 |
| ***S. mitis*** | 500 | 19.6 |
| ***C. albicans*** | 0.25 | 19.6 |
| ***C. krusei*** | 0.06 | 19.6 |
| ***C. tropicalis*** | 0.06 | 19.6 |
| ***C. glabrata*** | 0.125 | 19.6 |

**Duration of Antimicrobial Activity of Halo™ *in vitro*: Determination of Post-Antimicrobial effect (PAE)**

Post antimicrobial effect (PAE) is defined as suppression of microbial growth that persists after limited exposure to an antimicrobial agent. Having a longer PAE is considered advantageous for antimicrobial agents as it allows for persistent inhibition of microbial growth, and may affect dosing regimens since agents with long PAEs may need less frequent administration than those with short PAEs. Previous studies have described PAE for both antibacterial and antifungal agents [[27-30](#_ENREF_27)]. We evaluated the PAE of Halo™ against oral bacteria (*S. pneumonia*, *S. sanguis*, *S. gordonii* and *S. aureus*). Briefly, cells were exposed to Halo™ (at a concentration equal to the MIC) for 1 min followed by three washes to remove residual drug. Plates were incubated at 37 °C and time taken for the cells to regrow was determined. PAE was calculated as the time difference required for a culture to grow 1 log_10_ following exposure to and removal of Halo™ at its MIC compared to the time required for 1 log_10_ of growth in an unexposed control culture [[28](#_ENREF_28)]. PAE was expressed as the time (in hours) for which growth inhibition (%) was maintained by Halo, compared to untreated control.

| 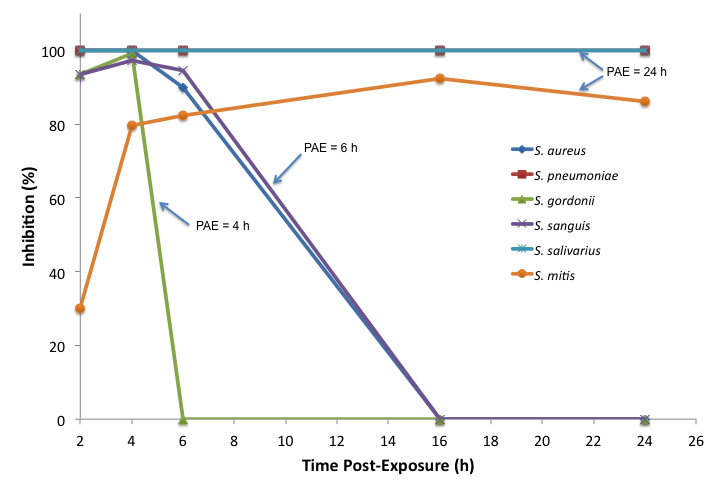 |
| --- |
| **Fig. 10. Post antimicrobial effect (PAE) of Halo against oral bacteria** |

As shown in Figure 10, the post-antimicrobial effect (PAE) of Halo ranged between 4 h and 24 h against *S. aureus*, *S. pneumonia*, *S. gordonii*, *S. sanguis*, *S. salivarius*, and *S. mitis*. These results show that Halo™ exhibits activity against oral bacterial for 4-24 h.

**Effect of Halo on bacterial ultrastructure**

To determine the effect of Halo on the morphology of bacteria, we examined the ultrastructure of bacteria (*S. sanguis* and *S. oralis*) exposed to Halo compared to untreated controls using scanning electron microscope (SEM) as described previously [[31](#_ENREF_31)]. Briefly, cells were grown in medium in the absence (control) or presence of sub-MIC levels of Halo for 24 h. Next, cells were washed to remove residual agent, and dehydrated by passing through a series of alcohol solutions (10% to 100% alcohol), and processed for SEM analysis. Our results showed that unlike untreated controls which demonstrated healthy intact cells, cells exposed to Halo were deformed and had collapsed structures, with total destruction of cellular integrity (Fig. 11).

| 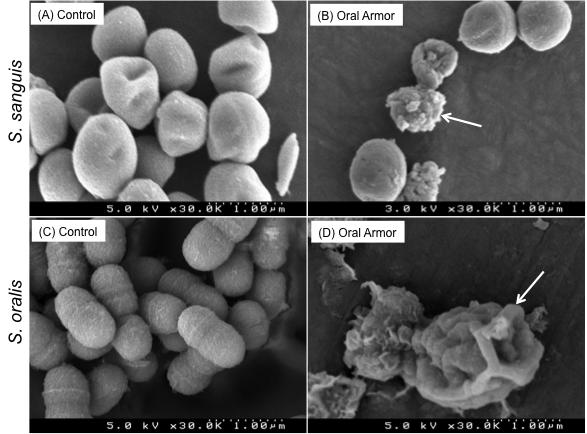  **Figure 11. Effect of Halo™ on bacterial ultrastructure.** Cells were grown in the absence (A-C) or presence (B-D) of Halo™, and analyzed by SEM. |
| --- |

**Exposure of bacteria to Halo™ inhibits cell growth: Time lapse microscopy**

To determine the inhibitory activity and duration for which Halo™ exposure remains effective against microbes, we performed a time-lapse analysis of cells exposed to this agent compared to bacteria untreated with this agent (controls). Bacteria were exposed to Halo™ for one minute, washed to remove any residual agent, and allowed to grow in a Petri-Dish containing fresh growth medium. Growth of bacteria at 37 °C was monitored for a 6-h period, and photomicrographs were taken every 20 min over the 6-h incubation period using a camera connected to the microscope. As shown in Figure 12, in contrast to the untreated control, where cells reached confluence by 6 h, cells treated with Halo™ failed to regrow during the same time period post-exposure. These results further confirmed that Halo™ possesses prolonged antimicrobial activity.

| 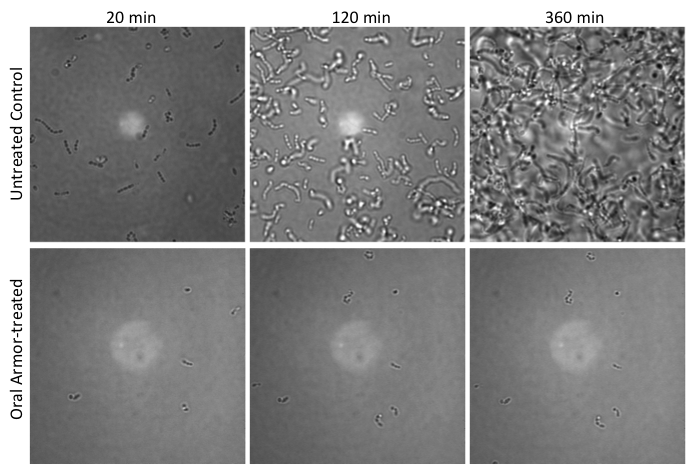  **Figure 12. Bacterial growth after a 1-min exposure to Halo™.** Images represent bacterial growth after 20 min, 120 min, or 360 min post-exposure. |
| --- |

**Activity against biofilms**

Since oral microbes often manifest their infectious potential as biofilms (plaques), we also determined whether Halo™ possess anti-biofilm activity. We used the method developed by our group earlier to evaluate the activity of Halo™ against biofilms formed by oral microbes (both bacteria and yeast) [[32](#_ENREF_32)].

| 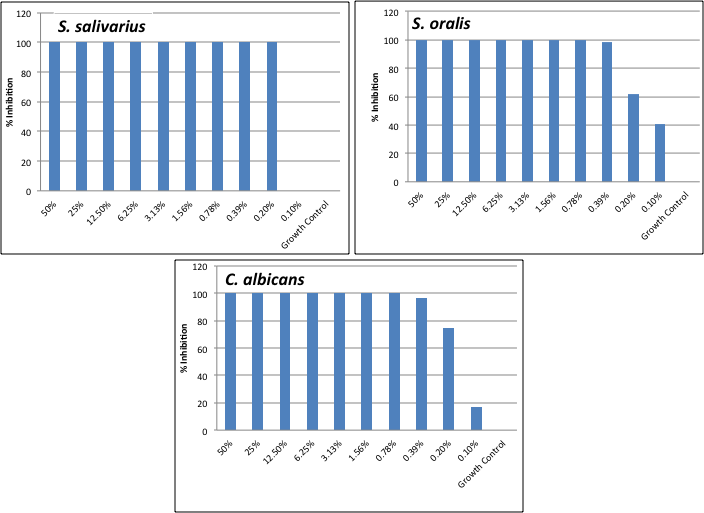 |
| --- |
| **Figure 13. Inhibition of biofilm growth by Halo™.** |

Our results (Fig. 13) showed that Halo™ inhibited bacterial and fungal biofilms, with an MIC of 0.2% against biofilms formed by *S. salivarius*, *S. oralis*, or *C. albicans*.

**Barrier Function of Halo™**

To evaluate the ability of Halo™ to create a protective barrier on human mucosal tissues, we used an engineered human oral mucosa (EHOM) model, in collaboration with Dr. Rouabhia (Université Lavale, Quebec, Canada). The EHOM tissues simulate the oral mucosa and provide a robust system in which the effect of agents on mucosal tissues can be evaluated. EHOM layers were formed as described in our earlier publication [[33](#_ENREF_33), [34](#_ENREF_34)]. Halo was added to EHOM tissue in a well, and allowed to form a uniform layer on the tissues. These Halo™-covered tissues were then overlaid with standardized number of bacteria or fungi and incubated overnight. After the incubation period, the flow-through solution was collected from the bottom well, and cultured to detect the presence of microbes. As shown in Figure 14, flow-through solution of EHOM tissues treated with Halo™ did not contain any fungal cells, in contrast to the untreated control where profuse growth was observed. Similar results were observed for bacterial cells. This datais in agreement with our earlier findings (see above) with viruses where using a 0.02% concentration of Halo did not have a direct effect against influenza viruse, while coating cultured cells with this formulation at the same concentration did protect the cells against virus infection. These results demonstrate that Halo acts, in part, by forming a protective barrier on the oral mucosa, which in turn prevents microbial attachment and microbial penetration in host tissue.

| 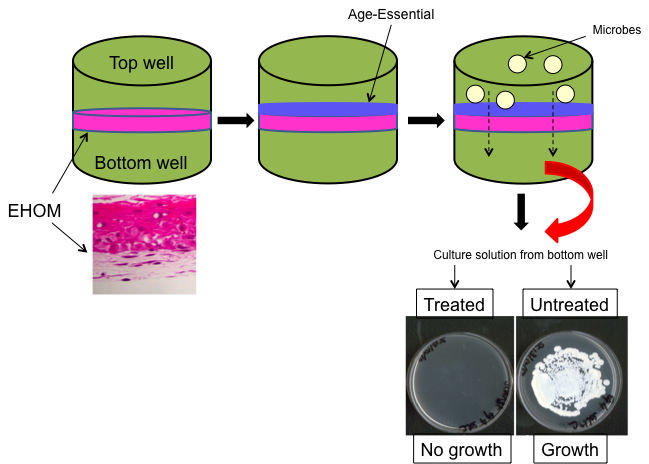 |
| --- |
| **Figure 14. Halo™ Forms a Protective Barrier on the Oral Mucosa** |

**Experimental Design and Methods**

**Overview**

This is a pilot study of the safety, tolerability, and effectiveness of Halo to prevent acute upper respiratory illness and respiratory virus infections. This study will be conducted at one site (University Hospitals Case Medical Center) in healthy adults during the upcoming respiratory virus season. The intervention will be with Halo, a commercial product which is FDA-approved for the treatment of xerostomia. The placebo will consist of purified sterile water without CPC - the active antiseptic. This placebo was chosen as the Halo formulation without CPC serves to act as a barrier to attachment of oral pathogens, and as such is an important contributing factor to its antimicrobial activity (see above). The use of preservative could be problematic as there may be some anti microbial activity. Without the use of preservatives, the product will be refrigerated except for administration.

Male and female participants 18-45 years of age will be recruited and monitored for the development of, duration, and severity of clinical symptoms and signs consistent with acute respiratory disease (defined below) captured daily through diaries, and PCR confirmation of important respiratory viruses including influenza, rhinoviruses, adenoviruses, and respiratory syncytial virus during episodes of acute respiratory disease during the length of the study will be undertaken. Secondary objectives will assess the tolerance, acceptability and adherence to Halo as well as the change in the bacterial (oral streptococci and Group A streptococcus) and fungal microflora in the oropharynx. School or work absenteeism, visits to physicians’ offices, emergency departments and urgent care centers will also be captured. Conventional cultures for these bacterial and fungal organisms will be pursued (see below). Throughout the study period, the safety, tolerability, acceptability and adherence to study products will be assessed.

**Selection and enrollment of participants**

**Study Enrollment and Withdrawal**

One hundred two healthy male and female participants, ages 18-45 will be enrolled in two groups. It is estimated that 204 subjects will need to be screened (2:1 screened to eligible) to enroll 102 subjects. At screening/enrollment, subjects will be stratified as to whether they received inactivated or live influenza virus vaccination. Subjects will be recruited through customary IRB-approved methods. Children, pregnant women, prisoners and other vulnerable individuals will not be enrolled.

Screening will begin with a brief IRB-approved telephone screen. Information about the study will be presented to potential subjects. The telephone script will include the inclusion/exclusion criteria. Appointments will be made for subjects who are interested in the study at the Case Western Reserve University Dental School Room 3080 for further screening/enrollment procedures and protocol-specific information.

Study retention strategies will include education and explanations of the study schedule and procedures during enrollment. In addition, reimbursements will be dispersed at specified time points during the study with the amount contingent on completion of study procedures. Participants will be reminded before visits, and the clinical research study staff will contact participants who miss appointments to determine if they wish to return for further evaluation.

**Inclusion Criteria**

1. Signed informed consent

2. Ages between 18-45 years, inclusive

3. Non-tobacco/nicotine use (at least 3 months)

4. BMI of 17-35 kg/m^2^ inclusive

5. Able to comply with all study procedures and follow-up

6. Healthy on the basis of screening medical exam, blood chemistries, hematology,

and serum pregnancy tests for females (at screening )

7. Non-pregnant or breast-feeding

**Exclusion Criteria:**

1. History of current use of narcotics, or recreational drug use.
2. Use of > 7 alcoholic beverages in 1 week

2. Any acute illness such as asthma, cardiovascular, respiratory, hepatic, renal,

hematologic, gastrointestinal (including eating disorder), endocrine/metabolic,

neurologic, dermatologic, or psychiatric disease.

3. Treatment with another investigational product in the past 30 days

4. Immunocompromised status including HIV by history

5. Use of any other intra-oral product (especially antiseptic).

6. Intended use of mouthwash during study duration

7. Laboratory (screening) of creatinine > 1.5 X ULN, hemoglobin < 10.5 g/dL, platelet count ≤ 99,000/mm^3,^ absolute neutrophil count ≤1200/ mm^3^, serum glucose ≥ 150 mg/dl (Subject can enroll prior to receiving lab results. Subject will be contacted if lab results are an exclusion and discontinue from study)

8. Use of any systemic antibiotic or antifungal within 30 days of screening

9. Prior or current history of seasonal allergies, chronic sinusitis or rhinitis

10. Allergy to any component of Halo

11. Oral lesions

12. Dentures

13. Dental Treatment currently in progress (implants, root canals, complex

restorative procedures)

**Study Outcome Measures**

The **primary study objective** is to establish whether Halo will decrease the frequency, duration, and severity of acute respiratory disease and decrease the detection of respiratory viruses by multiplex PCR. After enrollment, subjects will be seen at scheduled monthly visits (+/- 7 days), 1-2 months after study completion and at interim visits when they develop a syndrome consistent with an acute upper respiratory disease. An acute upper respiratory disease will be defined as having fever to > 37.8°C and a non-productive cough and 2 or more of the following symptoms:

- Sore throat
- Rhinorrhea
- Sinus congestion
- Malaise

Patients will be provided with diaries (RedCap) to note any illness especially acute respiratory disease and to record the duration of each sign or symptom. Subjects will also be asked to take their temperature at 5p.m. each day and record their temperatures in the diaries. At interim visits related to the development of acute upper respiratory illness oropharyngeal swabs will be obtained for bacterial and fungal culture as well as respiratory virus multiplex PCR.

**Multiplex RT-PCR:** Following collection, an aliquot of the clinical sample will be placed in viral freezing media and stored at -80 degrees. Samples will be placed in 2ml glass collection tubes at a 1:1 ratio of clinical sample to viral freezing media. RNA from each clinical specimen will be extracted with the QiaAmp Viral RNA Mini Kit (Qiagen Inc., Valencia, CA) according to the manufacturer’s protocol. Extracted RNA will be stored at -80 degrees until processing.

Primers and probe sets for multiplex real-time RT-PCR detection of respiratory tract viruses have already been described [[35](#_ENREF_35)]. We will screen influenza positive samples under conditions as described with the exception that chlamydia and mycoplasma primer sets will be omitted. In addition, screening reactions for recently recognized viruses will be performed separately using real time primer sets as previously described [[36](#_ENREF_36), [37](#_ENREF_37)].

Microbial cultures will be performed from oral swabs obtained from enrolled study participants. Briefly, collected swabs will be vortexed in 1 mL saline, and a calibrated 10 µL inoculation loop will be used to spread the resulting suspension (and its serial dilutions) onto agar plates specific for aerobic or anaerobic bacteria (blood agar), or fungi (Sabouraud dextrose). Plates will be incubated at 37 °C overnight and the resulting CFUs will be counted to determine the microbial load.

In terms of safety and tolerance of Halo and placebo, the oropharynx and gingiva will be examined for erythema and ulcerations during each scheduled visit. This will not be pursued at the interim visits when presenting with an acute upper respiratory disease as mucosal changes are likely to be present related to these syndromes. Subjects will be queried about any intraoral or systemic adverse reactions. Toxicity will be graded according to modification of the National Institutes of Health, National Institute of Allergy and Infectious Diseases, DMID Adult Toxicity Tables provided in Appendix 1.

Subjects will keep a daily diary of oral temperature, product usage adherence, typical cold and flu symptoms that may occur, school or work absenteeism, and visits to physicians’ offices, emergency departments and urgent care centers using REDCap electronic data capture tools hosted at University Hospitals Case Medical Center. REDCap (Research Electronic Data Capture) is a secure, web-based application designed to support data capture for research studies. Following the completion of the study, participants will be asked to take a short survey through the same REDCap system in order to assess the acceptability of the product and to gather information about the volunteers’ willingness to continue to use the product beyond the study parameters and if they would recommend the product to others. Finally, to further assess adherence to the study, product bottles will be counted.

**Randomization and Withdrawal Procedures**

The randomization list will be generated and provided to the study pharmacist. The study blind (except for the Study Pharmacist) will be maintained by all study personnel. The investigators may request that the blind be broken for medical reasons.

Each subject will be encouraged to complete the full course of the intervention assignment and study assessments. However, it is understood that the subject may discontinue study participation at any time for any reason. In addition, subjects should be encouraged to return to the unit within 28 days after dropout for additional safety assessments. The reason for early withdrawal must be documented in the CRF and in the source documents. Subjects are free to withdraw from the study at any time for any reason. Subjects should normally be withdrawn from the trial if a serious adverse event (SAE) occurs. Exceptions must be discussed with the Sponsor.

Subjects **must** be withdrawn from the trial if:

1. They withdraw their consent;
2. The investigator considers it in the best interest of the subject that he or she is withdrawn;
3. They experience a grade 3 or 4 adverse event (AE) (Appendix 1)

The reason for any subject’s discontinuation and the date of withdrawal will be recorded in the subject’s CRF. The subject’s CRF, which will be completed up to the point of withdrawal. The study report will include reasons for subjects’ withdrawals as well as details relevant to the subjects’ withdrawals. Any subject withdrawn from the trial prior to completion will undergo all procedures indicated in this protocol as being scheduled to occur at discharge or upon early withdrawal, subject to his/her consent. Any subject withdrawn due to an adverse event (whether serious or non-serious) or any clinically significant abnormal laboratory test value will be evaluated by the Principal Investigator or a monitor (see Key Personnel), and will be treated and followed up until the symptoms or values return to normal or acceptable levels, as judged by the Principal Investigator.

Relevant post-study procedures will be performed, wherever possible, on subjects who elect to withdraw. If a subject is withdrawn from participation in the study at any time at his or her request or as determined by the Study Investigators, the reason(s) for discontinuation shall be documented thoroughly in the source documents and CRFs. If a subject is discontinued because of an adverse event, this event will be followed until it is resolved or the subject is clinically stable and will also be documented in the source documents and CRFs.

Subjects who receive any amount of study product and who subsequently withdraw or are withdrawn from the study will be encouraged to continue Follow-up (with subject’s consent) for safety. The subject will be asked for permission to continue scheduled evaluations and complete an end-of study evaluation. If an AE or SAE has occurred, every effort will be made to undertake protocol-specific safety follow-up procedures, and the subjects will be encouraged to receive appropriate care until the signs and symptoms or laboratory toxicities resolve.

**INVESTIGATIONAL PRODUCTS**

**Study Product Description**

Halo is a proprietary formulation in which a high concentration of a polyvinyl pyrollidone backbone polymer is incorporated into an anionic polymer solution as well as an antimicrobial agent, cetylpyridinium chloride (CPC) at 0.1%. The formulation has high mucoadhesion properties and prolonged retention time in the oral cavity (21). Cetylpyridinium chloride like chlorhexidine is a quarternary ammonium salt antiseptic. Due to its chemical structure, CPC demonstrates significant antiviral activity especially against respiratory viruses (discussed in Preliminary Studies). Toxicology studies have shown no evidence of acute or chronic toxicity in animal models or irritant effect or in human use for the treatment of xerostomia (ref). In this study the placebo will consist of purified sterile water without CPC - the active antiseptic. This placebo was chosen as the Halo formulation without CPC serves to act as a barrier to attachment of oral pathogens, and as such is an important contributing factor to its antimicrobial activity (see above). The use of preservative could be problematic as there may be some anti microbial activity. Without the use of preservatives, the product will be refrigerated except for administration.

**Safety:**

Halo is an oral spray that is intended to provide reduce the microbial load in the oral cavity which may help protect against infections, including respiratory disease. The product has been formulated with a combination of lubricating/hydrating polymers and humectants. It contains 0.1% cetylpyridinium chloride as the active ingredient as well as the excipients, 35% glycerin copovidone, flavor, methylparaben, propylparaben, PEG-60 hydrogenated castor oil, sodium benzoate, sodium saccharin, water, xanthan gum and xylitol. Halo Spray is dosed from a conventional pump spray bottle without the aid of propellants. The consumer is directed to hold the spray bottle upright and to spray six times into the mouth without rinsing or spitting out. The recommended dosage is as required 3 times or 9 sprays per day.

**Toxicological Risk Analysis**

A safety assessment of this formulation was conducted with reference to BSEN ISO 10993, biological assessment of medical devices. This formulation was evaluated for acute oral toxicity, mucosal irritation, sensitization, subchronic toxicity, and genotoxicity potential. Evaluation of this formulation also took into consideration individuals with compromised immune systems as it is intended for use by individuals with medication-induced xerostomia as well as individuals with Sjogren's syndrome. Results of these evaluations indicate the product has an acceptable safety profile and poses no significant toxicological risk to individuals in these subpopulations under the intended conditions of use. The formulation consists of ingredients with a long history of safe use in oral healthcare products. With the exception of the flavor ingredients, Optamint 828653, Mouthwatering Liquid 15803210 and Optamint Optaflow225448, an adequate safety margin can be calculated to exist between the maximum daily exposure to each ingredient and exposures that can elicit adverse effects. All ingredients are of approved pharmaceutical grade (United States Pharmacopoeia and National Formulary, European Pharmacopoeia and British Pharmacopoeia) or comply with an internal standard. This formulation is composed of ingredients that are free of animal-origin and genetically modified-origin materials.

This formulation contains ingredients similar to other products intended to provide relief from the symptoms of dry mouth which have been shown to have acceptable safety profiles. There is no evidence to suggest additive or synergistic effects may arise following use of this product formulation.

The active ingredient, cetylpyridinium chloride has been used in mouth rinses in the United States since 1940. Cetylpyridinium chloride 0.025 percent to 0.1 percent has been marketed nationally in several products. Products containing cetylpyridinium chloride have also been marketed internationally. The more than 55-year U.S. marketing history is significant with respect to the ingredient's safety (Oral Health Care Drug Products for Over-the-Counter Human Use; Antigingivitis/ Antiplaque Drug Products; Establishment of a Monograph; Proposed Rules.(Federal Register, May 29,2003, Volume 68, Number 103: 32231-32287). Therefore, the risk of systemic toxicity following oral administration of cetylpyridinium chloride at the concentration used in this spray formulation is considered minimal.

Safety data on the flavor ingredients are available and are considered adequate to support their use in this formulation. The flavors used consist of mixtures of essential oils and other flavor ingredients, all of which are commonly used flavor constituents at levels similar to those used in current market products. Although actual safety margins cannot be calculated for these flavors, due to the proprietary nature of their composition, the suppliers evaluated the flavors for safety when used in this type of dosage form at the same concentrations at which they are used in this formulation and concluded that the flavors satisfy, according to the current state of knowledge, the safety requirements for the intended application under normal and reasonably foreseeable conditions of use. The flavor suppliers also declared that the flavors are acceptable for use in foods and cosmetics by their compliance with the provisions of Directives 88/388/EEC and 76/768/EEC and that the components of the flavors comply with an applicable regulation of FDA and/or are designated as being generally recognized as safe. As such these flavors are considered safe for inclusion in this formulation.

All of the remaining ingredients in this formulation are of very low toxicity and have a long history of safe use in oral healthcare products marketed in the United States and Europe. Their presence in this formulation, in the amounts specified, presents no significant toxicological risk.

This formulation contains trace amounts of the ICH Class 2 solvents methanol, 1,4-dioxane, ethylene glycol, toluene and pyridine as well as trace amounts of the ICH Class 3 solvents methyl ethyl ketone, ethyl acetate, ethanol, 2-propanol, acetone and 1-propanol. The residual concentrations of these solvents in this formulation are less than the concentration limits defined for each in the ICH guidance on residual solvents (CPMP/ICH/283/95) and the potential maximum daily exposure to each of these solvents from use of this formulation therefore conforms to the recommendations o f Option 1 o f the ICH guidance.

Assessment of potential hazard risks associated with use of Halo Spray reveals no significant safety issues. The formulation is similar to other products intended to provide relief from the symptoms of dry mouth which have been shown to have acceptable safety profiles. Proper use of this formulation as a mouth spray is not anticipated to be associated with any potential hazards. Although improper use of this formulation may be associated with potential hazards, the instructions and warning statements included in the product labeling are considered adequate to render the risk of such hazards acceptable. In summary, toxicological evaluation of Halo Spray concludes that the product does not present any significant toxicological risks to individuals when used as directed.

**Storage**

Halo is stable at room temperature. It can also be stored in the refrigerator (4^O^ C). At both storage temperatures, it was shown that the physical and microbiological are maintained. The Placebo should be stored in the refrigerator (4^O^ C). To maintain the blind both Halo and Placebo will be stored in the refrigerator (4^O^ C).

**Labeling:**

Halo is an OTC Drug (Antiseptic, Statement of Identity). It has an OTC Oral Healthcare Monograph active (cetylpyridinium chloride). It kills germs and may help guard against infections.

**Administration**

Based upon the preliminary studies that assessed the duration of the antimicrobial effects of Halo as reported in the preliminary studies and feasibility, subjects assigned to both study arms will be asked to spray the product intra-orally 3 times daily (Three sprays per use, total = 9 sprays per day). Although it is recognized that respiratory viruses most often and initially invade the nasopharynx (ref), it is still the case that the oropharynx is also involved in the pathogenesis of disease and there may be diffusion to the nasopharynx of orally administered product given the anatomic proximity of these anatomic sites.

As noted above, the study products will be supplied in 30 mL bottles to participants. Product should preferably be administered 30 minutes after any meal or brushing of teeth throughout the day and right before retiring. In between uses, the study product will be kept at room temperature. Subjects will be asked to retain bottles of all study products to be brought to scheduled visits to be counted by study personnel as a measurement of adherence. The date of initial use and the final use will be recorded on each study bottle.

**Clinical/Laboratory Evaluations and Study Schedule**

**Screening/Enrollment (See Appendix 2, Schedule of Events)**

Written informed consent will be obtained before any clinical evaluations are performed. The study will consist of 2 groups randomly assigned to Halo or matching placebo. The study visits will be conducted in the CWRU School of Medicine Room 3080. Screening and Enrollment will be performed at the same visit, a complete medical history and physical exam will be performed by study clinicians along with, serum chemistries, CBC with differential count, and a serum pregnancy test for women. Randomization into the study will be undertaken when it is determined that the subject has met all study inclusion criteria and has no exclusion criteria other than possible lab results. Since the screening and enrollment will be conducted at the same visit, subjects will begin the study prior to results of labs. If the labs result in an exclusion criteria the subject will be notified to discontinue and return unused study product. As noted in the safety section:. the study product has an acceptable safety profile and poses no significant toxicological risk to individuals. The formulation consists of ingredients with a long history of safe use in oral healthcare products.

A posterior pharyngeal and nasal swab will be obtained to ascertain baseline bacterial and fungal organisms and a multiplex PCR for respiratory viruses. Study product (after randomization) will be provided until the next scheduled visit, study procedures will be again reviewed, contact information for reaching study personnel will be provided, and study diaries will be distributed. Appointments for the 1^st^ monthly evaluation will be made.

**Scheduled Follow-up Visits**

At these visits, an interval medical and concomitant medication history will be obtained. Solicited and unsolicited adverse reactions will be ascertained. The study diaries will be reviewed with participants. An oropharyngeal exam will be conducted by study clinicians and a fuller symptom-directed physical exam will be performed if indicated. The intra-oral examination will be performed by utilizing the diagram provided in Appendix 3. A posterior pharyngeal and nasal swab will be obtained at each visit to ascertain bacterial and fungal organisms and a multiplex PCR for respiratory viruses. The enrollment and interim visit pharyngeal and nasal swabs will be analysed in real time, all other swabs will be stored for future use determined by funding. Study bottles will be collected and counted. Study product will be provided until the next scheduled visit, study procedures will be again reviewed, and contact information for reaching study personnel will be revisited. Appointments for the next monthly evaluation will be made.

**Final Study Visit**

This study visit will be conducted within 2 months after completing the last dose of Halo/Placebo. At this visit, an interval medical and concomitant medication history will be obtained. Solicited and unsolicited adverse reactions will be ascertained. An oropharyngeal exam will be conducted by study clinicians and a fuller symptom-directed physical exam will be performed if indicated. The intra-oral examination will be performed by utilizing the diagram provided in Appendix 3.

**Interim Visits**

Subjects will be instructed to contact study personnel in the event they develop an acute upper respiratory disease as defined above within 72 hours of onset of symptoms and signs (this allows for weekends and holidays). At these visits, an interval medical and concomitant medication history will be obtained. Solicited and unsolicited adverse reactions will be ascertained. An oropharyngeal exam will be conducted by study clinicians and a fuller symptom-directed physical exam will be performed if indicated. If pharyngitis with exudate is observed or suspected, a rapid group A streptococcal (GAS) screen will be performed. Subjects positive for Group A streptococcal infection will be given a prescription for appropriate antibiotics and a referral for follow-up care as needed. If individuals present with symptoms and signs of acute influenza fever, sore throat, myalgias, headache and chills, they will be tested for influenza and prescribed Tamiflu as needed. A posterior pharyngeal and nasal swab will be obtained to ascertain baseline bacterial and fungal organisms and a multiplex PCR for respiratory viruses. Any visit to a physician’s office, emergency department, urgent care center or the need for hospitalization will be recorded. Subjects will be carefully instructed to record the duration of each symptom and sign to be recorded in the study-supplied diaries.

**Statistical Analysis**

**Sample Size Calculations**

Initially, primary outcomes in the control group vs. the treatment group will be compared using the t-test for independent samples, and this technique was used to estimate sample sizes. Specifically, sample size calculations were carried out assuming that the two groups will be of equal size and that the random assignment will be balanced. Further, it was assumed that an average of 2 events would occur in the control group, contrasted against an average of 1.5 events in the treated group (SD = 1), and that the average duration of illness would be 4 days and 3 days in the control and treated groups, respectively (SD = 2). Taking alpha to be 0.05, a sample size of 23 per group would allow the detection of a 25% difference in primary outcomes between the two groups with 80% power. The sample size was increased to 51 per group to account for potential losses to follow-up.

**Randomization**

Based on whether the enrolled study participants have received any type of flu shot within the past 3 months, the participants will be classified as influenza vaccine recipients and non-recipients. Within each category, they will be randomly assigned to the control group or treatment group. The assignment will be balanced so that equal numbers are assigned to each group.

**Interim Analysis**

We will carry out an interim analysis when all the patients recruited have been followed for one and a half months. Since this is a pilot study, this analysis will be carried out at a stringent level of significance.

**Statistical Analysis**

Standard techniques of descriptive statistics will be used to compare demographics and other patient characteristics in the two treatment groups. Since an additional focus of this pilot study is to obtain accurate estimates of the moments of the distributions of the outcome variables, confidence intervals will also be provided. Descriptive summaries of the number of events and duration of infection will be generated for the two groups. As stated above, the t-test will be used to compare estimates of the average number of events per person as well as the average duration of illness between the two groups. To the extent that the small sample size allows it, we will utilize regression techniques for count data, using SAS GENMOD, to model the number of events per person adjusting for relevant covariates.

**References**

1 Mathers C, Fat DM, Boerma JT, World Health O. *The global burden of disease : 2004 update*. Geneva, Switzerland :: World Health Organization, 2008.

2 Graham NM. The epidemiology of acute respiratory infections in children and adults: a global perspective. *Epidemiol Rev* 1990; **12**: 149-78.

3 Merrill CT EA. Hospitalization in the United States, 2002. Rockville, MD: Agency for Healthcare Research and Quality. *HCUP Fact Book No 6* 2005; **Publication number 05-0056**.

4 van den Hoogen BG, de Jong JC, Groen J, et al. A newly discovered human pneumovirus isolated from young children with respiratory tract disease. *Nat Med* 2001; **7**: 719-24.

5 van der Hoek L, Pyrc K, Jebbink MF, et al. Identification of a new human coronavirus. *Nat Med* 2004; **10**: 368-73.

6 Esper F, Weibel C, Ferguson D, Landry ML, Kahn JS. Evidence of a novel human coronavirus that is associated with respiratory tract disease in infants and young children. *J Infect Dis* 2005; **191**: 492-8.

7 Fouchier RA, Hartwig NG, Bestebroer TM, et al. A previously undescribed coronavirus associated with respiratory disease in humans. *Proc Natl Acad Sci U S A* 2004; **101**: 6212-6.

8 Woo PC, Lau SK, Chu CM, et al. Characterization and complete genome sequence of a novel coronavirus, coronavirus HKU1, from patients with pneumonia. *J Virol* 2005; **79**: 884-95.

9 Allander T, Tammi MT, Eriksson M, Bjerkner A, Tiveljung-Lindell A, Andersson B. Cloning of a human parvovirus by molecular screening of respiratory tract samples. *Proc Natl Acad Sci U S A* 2005; **102**: 12891-6.

10 Gaynor AM, Nissen MD, Whiley DM, et al. Identification of a novel polyomavirus from patients with acute respiratory tract infections. *PLoS Pathog* 2007; **3**: e64.

11 Allander T, Andreasson K, Gupta S, et al. Identification of a third human polyomavirus. *J Virol* 2007; **81**: 4130-6.

12 Chua KB, Crameri G, Hyatt A, et al. A previously unknown reovirus of bat origin is associated with an acute respiratory disease in humans. *Proc Natl Acad Sci U S A* 2007; **104**: 11424-9.

13 Monto AS. Epidemiology of viral respiratory infections. *The American journal of medicine* 2002; **112 Suppl 6A**: 4S-12S.

14 Rakes GP, Arruda E, Ingram JM, et al. Rhinovirus and respiratory syncytial virus in wheezing children requiring emergency care. IgE and eosinophil analyses. *Am J Respir Crit Care Med* 1999; **159**: 785-90.

15 Papadopoulos NG, Moustaki M, Tsolia M, et al. Association of rhinovirus infection with increased disease severity in acute bronchiolitis. *Am J Respir Crit Care Med* 2002; **165**: 1285-9.

16 Arden KE, Nissen MD, Sloots TP, Mackay IM. New human coronavirus, HCoV-NL63, associated with severe lower respiratory tract disease in Australia. *J Med Virol* 2005; **75**: 455-62.

17 Esper F, Weibel C, Ferguson D, Landry ML, Kahn JS. Coronavirus HKU1 infection in the United States. *Emerg Infect Dis* 2006; **12**: 775-9.

18 Chidekel AS, Rosen CL, Bazzy AR. Rhinovirus infection associated with serious lower respiratory illness in patients with bronchopulmonary dysplasia. *Pediatr Infect Dis J* 1997; **16**: 43-7.

19 Kim JO, Hodinka RL. Serious respiratory illness associated with rhinovirus infection in a pediatric population. *Clin Diagn Virol* 1998; **10**: 57-65.

20 Malcolm E, Arruda E, Hayden FG, Kaiser L. Clinical features of patients with acute respiratory illness and rhinovirus in their bronchoalveolar lavages. *J Clin Virol* 2001; **21**: 9-16.

21 Surveillance for pediatric deaths associated with 2009 pandemic influenza A (H1N1) virus infection - United States, April-August 2009. *MMWR Morb Mortal Wkly Rep* 2009; **58**: 941-7.

22 Halasa NB. Update on the 2009 pandemic influenza A H1N1 in children. *Curr Opin Pediatr*; **22**: 83-7.

23 Thomas TL, Garland FC, Mole D, et al. Health of U.S. Navy submarine crew during periods of isolation. *Aviat Space Environ Med* 2003; **74**: 260-5.

24 Cross ER, Hermansen LA, Pugh WM, White MR, Hayes C, Hyams KC. Upper respiratory disease in deployed U.S. Navy shipboard personnel. *Mil Med* 1992; **157**: 649-51.

25 White DW, Feigley CE, McKeown RE, Hout JJ, Hebert JR. Association between barracks type and acute respiratory infection in a gender integrated Army basic combat training population. *Mil Med* 2011; **176**: 909-14.

26 Sanchez JL, Binn LN, Innis BL, et al. Epidemic of adenovirus-induced respiratory illness among US military recruits: epidemiologic and immunologic risk factors in healthy, young adults. *J Med Virol* 2001; **65**: 710-8.

27 Zhanel GG, Kirkpatrick ID, Hoban DJ, Kabani AM, Karlowsky JA. Influence of human serum on pharmacodynamic properties of an investigational glycopeptide, LY333328, and comparator agents against Staphylococcus aureus. *Antimicrob Agents Chemother* 1998; **42**: 2427-30.

28 Zhanel GG, Saunders DG, Hoban DJ, Karlowsky JA. Influence of human serum on antifungal pharmacodynamics with Candida albicans. *Antimicrob Agents Chemother* 2001; **45**: 2018-22.

29 Manavathu EK, Ramesh MS, Baskaran I, Ganesan LT, Chandrasekar PH. A comparative study of the post-antifungal effect (PAFE) of amphotericin B, triazoles and echinocandins on *Aspergillus fumigatus* and *Candida albicans*. *J Antimicrob Chemother* 2004; **53**: 386-9.

30 Clancy CJ, Huang H, Cheng S, Derendorf H, Nguyen MH. Characterizing the Effects of Caspofungin on Candida albicans, Candida parapsilosis, and Candida glabrata Isolates by Simultaneous Time-Kill and Postantifungal-Effect Experiments. *Antimicrob Agents Chemother* 2006; **50**: 2569-72.

31 Chandra J, Mukherjee PK, Ghannoum MA. In vitro growth and analysis of *Candida* biofilms. *Nat Protoc* 2008; **3**: 1909-24.

32 Mukherjee PK, Chandra J, Jurevic RJ, Ghannoum A, Ghannoum MA. Activity of Oasis™ and Biotene™ mouthwashes against microbial biofilms*IADR/AADR/CADR 89th General Session*. San Diego, CA: IADR/AADR, 2011.

33 Rouabhia M, Deslauriers N. Production and characterization of an *in vitro* engineered human oral mucosa. *Biochem Cell Biol* 2002; **80**: 189-95.

34 Mukherjee PK, Mohamed S, Chandra J, et al. Alcohol dehydrogenase restricts the ability of the pathogen *Candida albicans* to form a biofilm on catheter surfaces through an ethanol-based mechanism. *Infect Immun* 2006; **74**: 3804-16.

35 Brittain-Long R, Nord S, Olofsson S, Westin J, Anderson LM, Lindh M. Multiplex real-time PCR for detection of respiratory tract infections. *J Clin Virol* 2008; **41**: 53-6.

36 Mourez T, Bergeron A, Ribaud P, et al. Polyomaviruses KI and WU in immunocompromised patients with respiratory disease. *Emerg Infect Dis* 2009; **15**: 107-9.

37 Chow BD, Esper FP. The human bocaviruses: a review and discussion of their role in infection. *Clin Lab Med* 2009; **29**: 695-713.

APPENDIX 1: TOXICITY TABLE

**DMID Adult Toxicity Table, November 2007**

**ABBREVIATIONS:** Abbreviations utilized in the Table:

ULN = Upper Limit of Normal LLN = Lower Limit of Normal

R_x_ = Therapy Req = Required

Mod = Moderate IV = Intravenous

ADL = Activities of Daily Living Dec = Decreased

**ESTIMATING SEVERITY GRADE**

For abnormalities NOT found elsewhere in the Toxicity Tables use the scale below to estimate grade of severity:

**GRADE 1 Mild –** Transient or mild discomfort (< 48 hours); no medical intervention/therapy required

**GRADE 2 Moderate –** Mild to moderate limitation in activity - some assistance may be needed; no or minimal medical intervention/therapy required

**GRADE 3 Severe** **–** Marked limitation in activity, some assistance usually required; medical intervention/therapy required, hospitalizations possible

**GRADE 4 Life-threatening** – Extreme limitation in activity, significant assistance required; significant medical intervention/therapy required, hospitalization, or hospice care probable

**SERIOUS OR LIFE-THREATENING AEs**

ANY clinical event deemed by the clinician to be serious or life threatening should be considered a grade 4 event. Clinical events considered serious or life threatening include, but are not limited to: seizures, coma, tetany, diabetic ketoacidosis, disseminated intravascular coagulation, diffuse petechiae, paralysis, acute psychosis, and severe depression.

| **HEMATOLOGY** | | | | |
| --- | --- | --- | --- | --- |
|  | Grade 1 | Grade 2 | Grade 3 | Grade 4 |
| Hemoglobin | 9.5 - 10.5 gm/dL | 8.0 - 9.4gm/dL | 6.5 - 7.9 gm/dL | < 6.5 gm/dL |
| Absolute Neutrophil Count | 1000-1500/mm^3^ | 750-999/mm^3^ | 500-749/mm^3^ | <500/mm^3^ |
| Platelets | 75,000-99,999/mm^3^ | 50,000-74,999/mm^3^ | 20,000-49,999/mm^3^ | <20,000/mm^3^ |
| WBCs | 11,000-13,000/ mm^3^ | 13,000-  15,000 /mm^3^ | 15,000-  30,000/mm^3^ | >30,000 or <1,000 /mm^3^ |
| % Polymorphonuclear Leucocytes + Band Cells | > 80% | 90 – 95% | >95% |  |
| Abnormal  Fibrinogen | Low:  100-200 mg/dL  High:  400-600 mg/dL | Low:  <100 mg/dL  High:  >600 mg/dL | Low:  < 50 mg/dL | Fibrinogen associated with gross bleeding or with disseminated coagulation |
| Fibrin Split Product | 20-40 mcg/mL | 41-50 mcg/mL | 51-60 mcg/mL | > 60 mcg/mL |
| Prothrombin  Time (PT) | 1.01 - 1.25 x ULN | 1.26-1.5 x ULN | 1.51 -3.0 x ULN | >3 x ULN |
| Activated Partial Thromboplastin (APPT) | 1.01 - 1.66 x ULN | 1.67 - 2.33 x ULN | 2.34 - 3 x ULN | > 3 x ULN |
| Methemoglobin | 5.0 - 9.9 % | 10.0 - 14.9 % | 15.0 - 19.9% | > 20.0 % |

| **CHEMISTRIES** | | | | |
| --- | --- | --- | --- | --- |
|  | Grade 1 | Grade 2 | Grade 3 | Grade 4 |
| Hyponatremia | 130-135 mEq/L | 123-129 mEq/L | 116-122 mEq/L | < 116 mEq/L or abnormal sodium *with* mental status changes or seizures |
| Hypernatremia | 146-150 mEq/L | 151-157 mEq/L | 158-165 mEq/L | > 165 mEq/L or abnormal sodium *with* mental status changes or seizures |
| Hypokalemia | 3.0 - 3.4 mEq/L | 2.5 - 2.9 mEq/L | 2.0 - 2.4 mEq/L  or intensive replacement therapy or hospitalization required | < 2.0 mEq/L or abnormal potassium *with*  paresis, ileus, or life-threatening arrhythmia |
| Hyperkalemia | 5.6 - 6.0 mEq/L | 6.1 - 6.5 mEq/L | 6.6 - 7.0 mEq/l | > 7.0 mEq/L  or abnormal potassium *with* life-threatening arrhythmia |
| Hypoglycemia | 55-64 mg/dL | 40-54 mg/dL | 30-39 mg/dL | <30 mg/dL or abnormal glucose *with* mental status changes or coma |

| **CHEMISTRIES (continued)** | | | | |
| --- | --- | --- | --- | --- |
|  | Grade 1 | Grade 2 | Grade 3 | Grade 4 |
| Hyperglycemia  (nonfasting and no prior diabetes) | 116 - 160 mg/dL | 161- 250 mg/dL | 251 - 500 mg/dL | > 500 mg/dL or abnormal glucose *with* ketoacidosis  or seizures |
| Hypocalcemia  (corrected for albumin) | 8.4 - 7.8 mg/dL | 7.7 - 7.0 mg/dL | 6.9 - 6.1 mg/dL | < 6.1 mg/dL  or abnormal calcium *with* life threatening arrhythmia or tetany |
| Hypercalcemia  (correct for albumin) | 10.6 - 11.5 mg/dL | 11.6 - 12.5 mg/dL | 12.6 - 13.5 mg/dL | > 13.5 mg/dL or abnormal calcium *with* life  threatening  arrhythmia |
| Hypomagnesemia | 1.4 - 1.2 mEq/L | 1.1 - 0.9 mEq/L | 0.8 - 0.6 mEq/L | < 0.6 mEq/L or abnormal magnesium *with* life-threatening arrhythmia |
| Hypophosphatemia | 2.0 - 2.4 mg/dL | 1.5 -1.9 mg/dL or  replacement Rx required | 1.0 -1.4 mg/dL  intensive therapy  or  hospitalization required | < 1.0 mg/dL or abnormal phosphate *with* life-threatening arrhythmia |
| Hyperbilirubinemia (when accompanied by any increase in other liver function test) | 1.1 - <1.25 x ULN | 1.25 - <1.5 x ULN | 1.5 – 1.75 x ULN | > 1.75 x ULN |

| **CHEMISTRIES (continued)** | | | | |
| --- | --- | --- | --- | --- |
|  | Grade 1 | Grade 2 | Grade 3 | Grade 4 |
| Hyperbilirubinemia (when other liver function are in the normal range) | 1.1 - <1.5 x ULN | 1.5 - <2.0 x ULN | 2.0 – 3.0 x ULN | > 3.0 x ULN |
| BUN | 1.25 - 2.5 x ULN | 2.6 - 5 x ULN | 5.1 - 10 x ULN | > 10 x ULN |
| Hyperuricemia (uric acid) | 7.5 – 10.0 mg/dL | 10.1 – 12.0 mg/dL | 12.1 – 15.0 mg/dL | >15.0 mg/dL |
| Creatinine | 1.1 - 1.5 x ULN | 1.6 - 3.0 x ULN | 3.1 - 6 x ULN | > 6 x ULN or  dialysis required |

| **ENZYMES** | | | | |
| --- | --- | --- | --- | --- |
|  | Grade 1 | Grade 2 | Grade 3 | Grade 4 |
| AST (SGOT) | 1.1 - <2.0 x ULN | 2.0 – <3.0 x ULN | 3.0 – 8.0 x ULN | > 8 x ULN |
| ALT (SGPT) | 1.1 - <2.0 x ULN | 2.0 – <3.0 x ULN | 3.0 – 8.0 x ULN | > 8 x ULN |
| GGT | 1.1 - <2.0 x ULN | 2.0 – <3.0 x ULN | 3.0 – 8.0 x ULN | > 8 x ULN |
| Alkaline Phosphatase | 1.1 - <2.0 x ULN | 2.0 – <3.0 x ULN | 3.0 – 8.0 x ULN | > 8 x ULN |
| Amylase | 1.1 - 1.5 x ULN | 1.6 - 2.0 x ULN | 2.1 - 5.0 x ULN | > 5.1 x ULN |
| Lipase | 1.1 - 1.5 x ULN | 1.6 - 2.0 x ULN | 2.1 - 5.0 x ULN | > 5.1 x ULN |

| **CARDIOVASCULAR** | | | | |
| --- | --- | --- | --- | --- |
|  | Grade 1 | Grade 2 | Grade 3 | Grade 4 |
| Cardiac Rhythm |  | asymptomatic, transient signs, no  Rx required | recurrent/persistent;  symptomatic Rx required | unstable dysrhythmia; hospitalization and treatment required |
| Hypertension | transient increase  > 20 mm/Hg; no treatment | recurrent, chronic increase  > 20mm/Hg;  treatment required | acute treatment required; outpatient treatment or hospitalization possible | end organ damage or hospitalization required |

| **CARDIOVASCULAR** | | | | |
| --- | --- | --- | --- | --- |
|  | Grade 1 | Grade 2 | Grade 3 | Grade 4 |
| Hypotension | transient orthostatic hypotension with heart rate increased by <20 beat/min or decreased by <10 mm Hg systolic BP; no treatment required | symptoms due to orthostatic hypotension or BP decreased by <20 mm Hg systolic; correctable with oral fluid treatment | requires IV fluids; no hospitalization required | mean arterial pressure  <60mm/ Hg or end organ damage or shock; requires hospitalization and vasopressor treatment |
| Pericarditis | minimal effusion | mild/moderate asymptomatic effusion; no treatment | symptomatic effusion; pain; EKG changes | tamponade; pericardiocentesis or surgery required |
| Hemorrhage, Blood Loss | microscopic/occult | mild, no transfusion | gross blood loss;  1-2 units transfused | massive blood loss; > 3 units transfused |

| **GASTROINTESTINAL** | | | | |
| --- | --- | --- | --- | --- |
|  | Grade 1 | Grade 2 | Grade 3 | Grade 4 |
| Nausea | mild or transient; maintains reasonable intake | moderate discomfort; intake decreased significantly; some activity limited | no significant intake; requires IV fluids | hospitalization required; |
| Vomiting | 1 episode in 24 hours | 2-5 episodes in 24 hours | >6 episodes in 24 hours or needing IV fluids | physiologic consequences requiring hospitalization or requiring parenteral nutrition |
| Constipation | requiring stool softener or dietary modification | requiring laxatives | obstipation requiring manual evacuation or enema | obstruction or toxic megacolon |
| Diarrhea | mild or transient; 3-4 loose stools/day or mild diarrhea last < 1 week | moderate or persistent; 5-7 loose stools/day or diarrhea lasting >1 week | >7 loose stools/day or bloody diarrhea; or orthostatic hypotension or electrolyte imbalance or >2L IV fluids required | hypotensive shock or physiologic consequences requiring hospitalization |
| Oral Discomfort/Dysphagia | mild discomfort; no difficulty swallowing | some limits on eating/drinking | eating/talking very limited; unable to swallow solid foods | unable to drink fluids; requires IV fluids |
| **NEUROLOGICAL** | | | | |
|  | Grade 1 | Grade 2 | Grade 3 | Grade 4 |
| Neuro-Cerebellar | slight incoordination dysdiadochokinesis | intention tremor, dysmetria, slurred speech; nystagmus | locomotor ataxia | incapacitated |
| Psychiatric | mild anxiety or depression | moderate anxiety or depression; therapy required; change in normal routine | severe mood changes requiring therapy; or suicidal ideation; or aggressive ideation | acute psychosis requiring hospitalization; or suicidal gesture/attempt; or hallucinations |
| Muscle Strength | subjective weakness  no objective symptoms/ signs | mild objective  signs/symptoms  no decrease in function | objective weakness function limited | paralysis |
| Paresthesia (burning, tingling, etc.) | mild discomfort; no treatment required | moderate discomfort; non-narcotic analgesia required | severe discomfort; or narcotic analgesia required with symptomatic improvement | incapacitating; or not responsive to narcotic analgesia |

| **NEUROLOGICAL** | | | | |
| --- | --- | --- | --- | --- |
|  | Grade 1 | Grade 2 | Grade 3 | Grade 4 |
| Neuro-sensory | mild impairment in sensation (decreased sensation, e.g., vibratory, pinprick, hot/cold in great toes) in focal area or symmetrical distribution; or change in taste, smell, vision, and/or hearing | moderate impairment (mod decreased sensation, e.g., vibratory, pinprick, hot/cold to ankles) and/or joint position or mild impairment that is not symmetrical | severe impairment (decreased or loss of sensation to knees or wrists) or loss of sensation of at least mod degree in multiple different body areas (i.e., upper and lower extremities) | sensory loss involves limbs and trunk; paralysis; or seizures |

| **MUSCULOSKELATEL** | | | | |
| --- | --- | --- | --- | --- |
|  | Grade 1 | Grade 2 | Grade 3 | Grade 4 |
| Arthralgia  (joint pain) | mild pain not interfering with function | moderate pain, analgesics and/or pain interfering with function but not with activities of daily living | severe pain; pain and/or analgesics interfering with activities of daily living | disabling pain |

| **MUSCULOSKELATEL** | | | | |
| --- | --- | --- | --- | --- |
|  | Grade 1 | Grade 2 | Grade 3 | Grade 4 |
| Arthritis | mild pain with inflammation, erythema or joint swelling – not interfering with function | moderate pain with inflammation, erythema or joint swelling – interfering with function, but not with activities of daily living | severe pain with inflammation, erythema, or joint swelling – interfering with activities of daily living | permanent and/or disabling joint destruction |
| Myalgia | myalgia with no limitation of  activity | muscle tenderness (at other than injection site) or with moderate impairment of activity | severe muscle tenderness with marked impairment of activity | frank myonecrosis |

| **SKIN** | | | | |
| --- | --- | --- | --- | --- |
|  | Grade 1 | Grade 2 | Grade 3 | Grade 4 |
| Mucocutaneous | erythema; pruritus | diffuse, maculopapular rash, dry desquamation | vesiculation or moist desquamation or ulceration | exfoliative dermatitis, mucous membrane involvement or erythema, multiforme or suspected Stevens-Johnson or necrosis requiring surgery |
| **Induration** | **< 15mm** | **15-30 mm** | **>30mm** |  |
| **Erythema** | **< 15mm** | **15-30 mm** | **>30mm** |  |
| **Edema** | **< 15mm** | **15-30 mm** | **>30mm** |  |
| **Rash at Injection Site** | **< 15mm** | **15-30 mm** | **>30mm** |  |
| **Pruritus** | **slight itching at injection site** | **moderate itching at injection extremity** | **itching over entire body** |  |

| **RESPIRATORY** | | | | |
| --- | --- | --- | --- | --- |
|  | Grade 1 | Grade 2 | Grade 3 | Grade 4 |
| Cough | transient; no treatment | persistent cough;  treatment responsive | paroxysmal cough; uncontrolled with treatment |  |
| Bronchospasm, Acute | transient; no treatment;  70% - 80% FEV_1_  of peak flow | requires treatment; normalizes with bronchodilator;  FEV_1_ 50% - 70%  (of peak flow) | no normalization with bronchodilator;  FEV_1_ 25% - 50%  of peak flow; or retractions present | cyanosis: FEV_1_  < 25%  of peak flow or intubation necessary |
| Dyspnea | dyspnea on exertion | dyspnea with normal activity | dyspnea at rest | dyspnea requiring oxygen therapy |

| **SYSTEMIC** | | | | |
| --- | --- | --- | --- | --- |
|  | Grade 1 | Grade 2 | Grade 3 | Grade 4 |
| Allergic Reaction | pruritus without rash | localized urticaria | generalized urticaria; angioedema | anaphylaxis |
| Headache | Mild; no treatment required | transient, moderate; treatment required | severe; responds to initial narcotic therapy | intractable; requires repeated narcotic therapy |
| **SYSTEMIC** | | | | |
|  | Grade 1 | Grade 2 | Grade 3 | Grade 4 |
| Fever: oral | 37.7 - 38.5°C or  100.0 - 101.5°F | 38.6 - 39.5°C or  101.6 - 102.9°F | 39.6 - 40.5°C or  103 - 105°F | > 40°C or  > 105°F |
| Fatigue | normal activity reduced < 48 hours | normal activity decreased 25- 50% > 48 hours | normal activity decreased > 50% can’t work | unable to care for self |

| **ORAL** | | | | |
| --- | --- | --- | --- | --- |
| **Oral sites** | Grade 1 | Grade 2 | Grade 3 | Grade 4 |
| Mucosa  (buccal, labial, soft palate, and gingiva) (See Appendix 3 for more detail) | Erythema, and or superficial whitening and wrinkling of epithelium at contact site | Localized maculopapular lesions and or desquamation and ulceration | Diffuse vesiculation or desquamation and ulceration  (Lichenoid reaction) | Severe mucous membrane involvement or erythema, multiforme or suspected Stevens-Johnson or necrosis requiring surgery |
| Tongue | Erythema, altered papilla architecture or loss of papilla on dorsum of tongue | Localized desquamation, or ulceration localized | Diffuse vesiculation desquamation or ulceration  (Lichenoid reaction) | Severe mucous membrane involvement or erythema, multiforme or suspected Stevens-Johnson or necrosis requiring surgery |
| Floor of Mouth | Erythema, and or superficial whitening and wrinkling of epithelium at contact site | Localized desquamation or ulceration | Diffuse vesiculation or desquamation and ulceration  (Lichenoid reaction) | Severe mucous membrane involvement or erythema, multiforme or suspected Stevens-Johnson or necrosis requiring surgery |
| Palate | Erythema and or superficial whitening and wrinkling of epithelium at contact site | Localized maculopapular effects and or desquamation and ulceration | Diffuse vesiculization or desquamation and ulceration  (Lichenoid reaction) | Severe mucous membrane involvement or erythema, multiforme or suspected Stevens-Johnson or necrosis requiring surgery |
| Taste | Transient changes in taste perception  < 1 minute | Altered changes  Metallic, bitter last up to 1 hour | Lasting changes in taste >1 hour | Loss of taste |

**Appendix 2: Schedule of Events**

| **Procedure** | **Screening/Enrollment** | **Follow-up V1**  **(±7 days)** | **Follow-up V2**  **(±7 days)** | **Follow-up V3**  **(±7 days)** | **Final Study Visit**  **(within 2 months)** |
| --- | --- | --- | --- | --- | --- |
| Informed Consent | X |  |  |  |  |
| Medical History | X |  |  |  |  |
| Physical Exam | X |  |  |  |  |
| Serum Chemistries, CBC w/Diff, Serum Hcg | X |  |  |  |  |
| Review Inclusion/Exclusion | X |  |  |  |  |
| Interval Medical History | X |  |  |  |  |
| Posterior pharyngeal and Nasal Swab | X | X | X | X |  |
| Dispense Study Product | X | X | X |  |  |
| Study Diary Distributed | X | X | X |  |  |
| Review Study Procedures | X | X | X | X |  |
| Interval Medical & Concomitant Medication History |  | X | X | X | X |
| AE’s (solicited & unsolicited) |  | X | X | X | X |
| Review Study Diary |  | X | X | X |  |
| Oropharyngeal Exam |  | X | X | X | X |
| Symptom directed PE as indicated |  | X | X | X | X |
| Study Bottles collected counted |  | X | X | X |  |

## Appendix 3. Intra-oral diagram


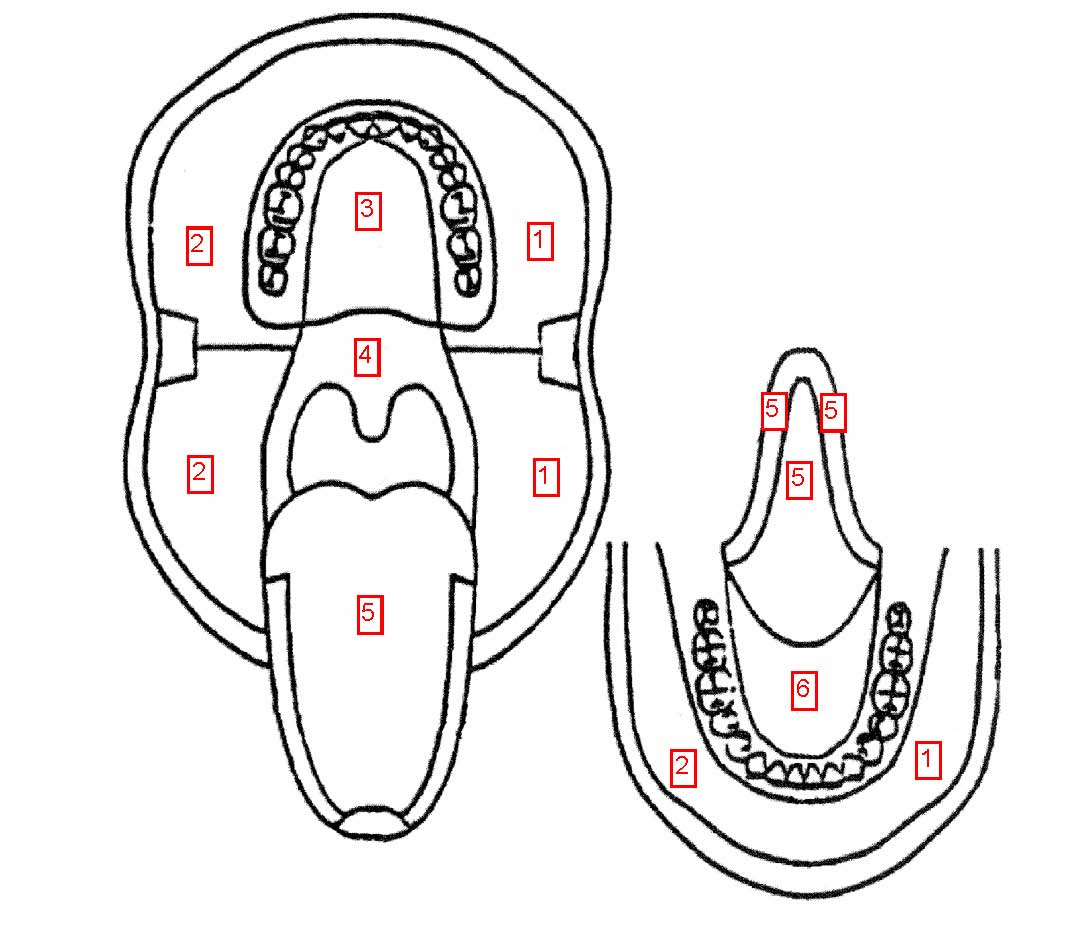


1) Left lower and upper labial mucosa and buccal mucosa

- - - 1. Right lower and upper labial mucosa and buccal mucosa
      2. Hard palate
      3. Soft palate
      4. Tongue (dorsum, lateral, and ventral)
      5. Floor of mouth

Oral Exam Results:

| Normal | Abnormal | Not Ascertained | If Abnormal, describe: |
| --- | --- | --- | --- |
